# Whole-Genome Analysis of the SHORT-ROOT Developmental Pathway in *Arabidopsis*

**DOI:** 10.1371/journal.pbio.0040143

**Published:** 2006-05-02

**Authors:** Mitchell P Levesque, Teva Vernoux, Wolfgang Busch, Hongchang Cui, Jean Y Wang, Ikram Blilou, Hala Hassan, Keiji Nakajima, Noritaka Matsumoto, Jan U Lohmann, Ben Scheres, Philip N Benfey

**Affiliations:** **1**Department of Biology and Institute for Genome Sciences and Policy, Duke University, Durham, North Carolina, United States of America; **2**Max Planck Institute for Developmental Biology, Tübingen, Germany; **3**Department of Molecular Cell Biology, Utrecht University, Utrecht, Netherlands; Howard Hughes Medical InstituteUnited States of America

## Abstract

Stem cell function during organogenesis is a key issue in developmental biology. The transcription factor SHORT-ROOT (SHR) is a critical component in a developmental pathway regulating both the specification of the root stem cell niche and the differentiation potential of a subset of stem cells in the *Arabidopsis* root. To obtain a comprehensive view of the SHR pathway, we used a statistical method called meta-analysis to combine the results of several microarray experiments measuring the changes in global expression profiles after modulating SHR activity. Meta-analysis was first used to identify the direct targets of SHR by combining results from an inducible form of SHR driven by its endogenous promoter, ectopic expression, followed by cell sorting and comparisons of mutant to wild-type roots. Eight putative direct targets of SHR were identified, all with expression patterns encompassing subsets of the native SHR expression domain. Further evidence for direct regulation by SHR came from binding of SHR in vivo to the promoter regions of four of the eight putative targets. A new role for SHR in the vascular cylinder was predicted from the expression pattern of several direct targets and confirmed with independent markers. The meta-analysis approach was then used to perform a global survey of the SHR indirect targets. Our analysis suggests that the SHR pathway regulates root development not only through a large transcription regulatory network but also through hormonal pathways and signaling pathways using receptor-like kinases. Taken together, our results not only identify the first nodes in the SHR pathway and a new function for SHR in the development of the vascular tissue but also reveal the global architecture of this developmental pathway.

## Abstract 2

Meta-analysis to combine the results of several microarray experiments reveals a new function for the transcription factor SHORT-ROOT in the development of vascular tissue.

## Introduction

**Figure 1:**
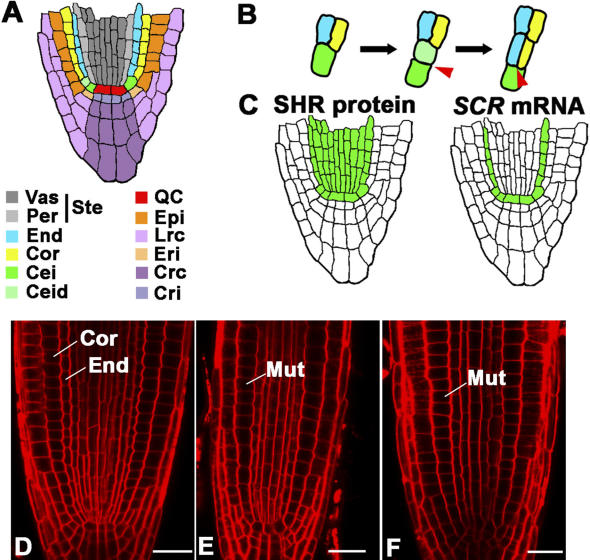
The *SHR* Pathway Is Essential for Stem Cell Specification and Radial Patterning of the Root (A) Schematic of a transverse section showing *Arabidopsis* root anatomy. Cei, cortex-endodermis initials; Ceid, cortex-endodermis initial daughters; Cor, cortex; Crc, columella root cap; Cri, columella root cap initials; End, endodermis; Epi, epidermis; Eri, epidermis-root cap initials; Lrc, lateral root cap; Per, pericycle; QC, quiescent center; Ste, stele; Vas, vascular cylinder. (B) Diagram of the cell divisions that form endodermis and cortex. The color code is as in (A). The two arrowheads indicate the transverse division of the Cei and the longitudinal division of the Ceid. (C) The SHR and SCR domain. The SHR protein (left) is present in the stele, the endodermis, the QC, the Cei, and the Ceid. *SCR* (right) is transcribed specifically in the QC, the Cei, and the Ceid. (D–F) Confocal section of roots from 5-d-old wild-type (D), *shr-2* (E), and *scr-4* (F). Cor, cortex; End, endodermis; Mut, mutant layer. Scale bars: 25 μm.

How stem cell populations are maintained and how their differentiation potential is regulated are key issues in developmental biology. In higher plants, stem cells are located in specialized structures called meristems [ 1]. In the root meristem of *Arabidopsis* ( Figure 1), the stem cells or initials surround a group of slowly dividing cells named the quiescent center (QC), which acts as a signaling center that positions and maintains the stem cell niche ( Figure 1A) [ 2, 3]. Stereotyped divisions of the initial cells generate the radial symmetry of the root, where concentric layers of epidermis, cortex, endodermis, and pericycle surround a central vascular cylinder, with the latter two tissues forming the stele. For example, cortex and endodermis (collectively named ground tissue) originate from the asymmetric division of a single initial cell in the meristem: the cortex/endodermis initial (CEI) ( Figure 1A and 1B) [ 4, 5]. This initial is first cleaved transversely, generating a new initial cell and a daughter cell. The CEI daughter cell then divides longitudinally, generating the first cells of the endodermal and cortical cell lineages.

SHORT-ROOT (SHR) is a putative transcription factor of the GRAS family and is a key component in a developmental pathway regulating the specification of the root stem cell niche as well as the radial patterning of the root in *Arabidopsis* [ 6– 8]. In *shr* mutants, a progressive disorganization of the QC is observed together with a loss of stem cell activity and a cessation of root growth. Mutation of the *SHR* gene also affects the development of the ground tissue and perturbs the radial pattern of the root. *shr* mutants do not undergo the longitudinal cell division of the CEI daughter cell, resulting in a single layer with only cortex attributes ( Figure 1D and 1E) [ 6, 7]. SHR is thus implicated in specification of the root stem cell niche, asymmetric division of the CEI, and endodermis fate specification. *SHR* mRNA is exclusively found in the stele but the SHR protein was shown to be present in all of the cells adjacent to the stele ( Figure 1C) [ 7, 8], suggesting that movement from the stele could allow SHR to act as a positional signal in stem cell specification and radial patterning.

Identification of the genes regulated by the SHR pathway is essential to fully understand SHR function in root development. However, our knowledge of this pathway is still limited and *SCARECROW (SCR)* is the only gene that has been demonstrated to be downstream of SHR [ 7, 8]. *SCR* encodes another member of the GRAS family and is expressed in all of the cells adjacent to the stele ( Figure 1C) [ 9]. In *scr* mutants, the asymmetric division of the CEI daughter also does not occur ( Figure 1D and 1F) and *SCR* was shown to be cell-autonomously required for QC specification and for the longitudinal cell division of the CEI daughter [ 9– 11]. The expression of *SCR* is strongly reduced in the *shr* mutant background, suggesting that SHR activity is necessary for full *SCR* expression [ 7]. Taken together, these observations show that *SCR* functions downstream of *SHR* in root stem cell niche specification and asymmetric cell division. However, expression of *SCR* in the QC of *shr* mutants cannot rescue QC function and only partially rescues stem cell maintenance [ 10], indicating that SHR function in stem cell specification is not limited to its requirement for activating *SCR*. Moreover, *SCR* is not involved in endodermis specification since the mutant ground tissue layer in *scr* plants has a mixed cortex/endodermis identity. A number of unidentified genes are thus expected to act downstream of *SHR* in parallel with *SCR*.

Genome-wide transcriptional analyses after modulation of transcription factor activity provide a powerful way to identify downstream genes. Although this allows for the screening of most of the genes in the genome, the large datasets require considerable effort to detect a signal among the noise [ 12, 13]. This is particularly true in multicellular organisms where genes can be expressed in small subsets of cells in an organ and transcriptional profiles are usually acquired from mixed tissue types [ 14– 16]. A common solution to find transcription factor targets in higher organisms is to generate several complementary datasets from tissues overexpressing the transcription factor of interest and from loss-of-function mutants or by doing time series experiments using inducible overexpression of the transcription factor [ 17– 22]. Analysis of consistency across experiments, using notably Venn diagrams, can then be used to minimize the rate of false-positives. The classical statistical method, called meta-analysis, combines the statistical information from several independent experiments and offers a way to maximize the use of the information available from each experiment. Meta-analysis was recently used to identify cancer signatures from tumor profiling experiments and was shown to decrease the false-positive rate by diminishing the artifacts associated with a single experiment [ 23– 25]. Importantly, meta-analysis was also shown to increase the statistical power of detecting small changes in gene expression using microarrays [ 23].

In this report, we used meta-analysis to study the SHR developmental pathway and implemented a new approach for the combination of multiple independent experiments to analyze developmental pathways in a multicellular organism. By borrowing strength from two other independent experiments, we were able to use an inducible form of SHR driven by the native *SHR* promoter to identify the direct targets of SHR. By doing so, we identified eight direct targets of SHR, one of which is the *SCR* gene. Binding of SHR to four of the eight promoter regions was confirmed in vivo using chromatin immunoprecipitation (ChIP) followed by quantitative real-time PCR. The expression patterns of the direct targets, determined using either “digital in situ” [ 26] or RNA in situ hybridization, show that the SHR protein regulates transcription in its entire domain, including the stele. This suggests that the SHR protein regulates the development of all of the tissues where it is present and was confirmed through further analysis of stele development in the *shr* mutant. To initiate the construction of a meaningful genetic network, we then used the meta-analysis approach to perform a global survey of the indirect targets regulated by SHR. Our analysis shows that SHR regulates not only a large transcription factor network but also hormonal pathways as well as signaling pathways using receptor-like kinases (RLKs). Taken together, our results thus not only identify the first nodes in the SHR pathway and a new function for SHR in the stele but also reveal the global architecture of this developmental pathway.

## Results

### Expression of an Inducible Form of SHR in Its Endogenous Domain Rescues Root Growth and Radial Patterning Defects in the *shr* Mutant

**Figure 2:**
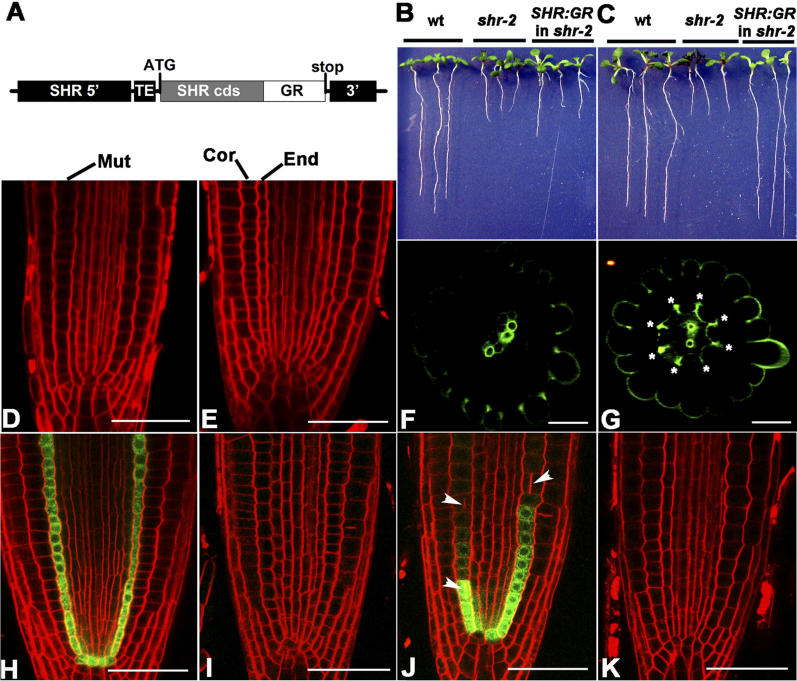
An Inducible Form of SHR Rescues Root Growth and Radial Patterning Defects in the *shr* Mutant (A) Structure of the *pSHR::SHR:GR* gene construct. DNA fragments are not drawn to scale. SHR 5′, 2.5-kb SHR 5′ upstream sequence; TE, tobacco etch virus translational enhancer sequence; SHR cds, SHR coding sequence; GR, steroid-binding domain sequence of the rat GR; 3′, nopaline synthase polyadenylation sequence. (B and C) Wild-type, *shr-2,* and *shr-2 pSHR::SHR:GR* 5-d-old seedlings germinated on a medium either without (B) or with (C) 10 μM Dex. (D and E) Confocal section of roots from 5-d-old *shr-2 pSHR::SHR:GR* plants germinated on a medium either without (D) or with (E) 10 μM Dex. Cor, cortex; End, endodermis; Mut, mutant. (F and G) JIM13 antibody staining of root transverse section from 5-d-old *shr-2 pSHR::SHR:GR* plants germinated on a medium either without (F) or with (G) 10 μM Dex. The stars indicate restoration of JIM13 staining in the ground tissue. (H–K) *pSCR::GFP* expression in roots of 5-d-old plants germinated on medium containing Dex and/or Cyc. (H) Root from untreated wild-type. (I) Root from untreated *shr-2 pSHR::SHR:GR* plants. (J) Root from *shr-2 pSHR::SHR:GR* plants treated with 10 μM Dex. The arrows indicate asymmetric cell divisions induced in response to the Dex treatment. (K) Root from *shr-2 pSHR::SHR:GR* plants treated with 10 μM Dex and 10 μM Cyc. Scale bars: 25 μm.

To produce the first reagent for the identification of SHR direct targets using meta-analysis, we generated a glucocorticoid inducible form of SHR ( Figure 2). Inducible versions of transcription factors have been widely used in plants for the identification of downstream targets [ 17, 18, 22, 27– 30]. However, while previous studies have relied on the use of a constitutive promoter, we sought to use a native promoter to minimize spurious effects linked to ectopic expression. The SHR protein was fused to the ligand-binding domain of the rat glucocorticoid receptor (GR) and expressed under the control of the 2.5-kilobase (kb) 5′ upstream region of SHR ( *pSHR::SHR:GR* construct: Figure 2A). The translational enhancer element (TE) from tobacco etch virus was also included to compensate for possible functional attenuation due to the protein fusion. The GR domain is expected to prevent a protein fused to it from entering the nucleus. This block can be released by treating with the synthetic glucocorticoid dexamethasone (Dex), thereby allowing the translocation of the fusion protein into the nucleus (reviewed in [ 31].

The *pSHR::SHR:GR* construct was introduced into the *shr-2* mutant background, which is a presumed null allele ( *shr-2 pSHR::SHR:GR* plants) [ 7]. As expected, *shr-2 pSHR::SHR:GR* plants looked identical to *shr-2* in the absence of Dex, in terms of root length and radial pattern ( Figure 2B and 2D). Upon germination of *shr-2 pSHR::SHR:GR* plants on a medium containing 10 μM Dex, we observed a complete rescue of root length, indicating a restoration of stem cell activity, and radial pattern ( Figure 2C and 2E and unpublished data). Transverse sections of plants grown in the presence of Dex were also used for immunostaining with the JIM13 antibody. The JIM13 antibody has been shown to specifically bind an arabinogalactan epitope found in endodermal and stele cells [ 32]. The immunostaining showed a Dex-dependent restoration of endodermal attributes ( Figure 2F and 2G).

To determine the time-frame for Dex treatment, we crossed the *shr-2 pSHR::SHR:GR* plants with a transgenic line expressing green fluorescent protein (GFP) under the control of the 2-kb 5′ upstream region of the *SCR* gene ( *pSCR::GFP* plants). As previously mentioned, *SCR* has been shown to be downstream of SHR [ 6, 7] and is expressed specifically in the endodermis, the CEI and daughter, and the QC ( Figure 2H) [ 9]. In the absence of Dex, no GFP expression was detected in 5-d-old *shr-2 pSHR::SHR:GR pSCR::GFP* plants ( Figure 2I), confirming previous results showing a decrease in *SCR* expression in *shr* mutants [ 7]. When these plants were transferred to a medium containing 10 μM Dex, we observed a strong accumulation of GFP after 6 h as well as a few asymmetric cell divisions (arrows in Figure 2J). Using quantitative real-time PCR (QRT-PCR), we found *SCR* mRNA levels to be similarly induced 6 and 24 h after Dex treatment in *shr-2 pSHR::SHR:GR* plants ( Figure S1). This suggests that SCR is already induced at its higher level 6 h after treatment. These observations indicate that at least some of the downstream targets of SHR are induced after 6 h of Dex treatment. They also confirm the results obtained with the JIM13 antibody and show that endodermal characteristics can be rapidly restored after treatment with Dex.

Fusion of GR to transcription factors that control floral organ development has been used to identify their direct targets through the concurrent short-term application of Dex with the translational inhibitor cycloheximide (Cyc) [ 22, 27, 30]. This allows for the transcription of immediate targets but presumably blocks those genes that are further downstream in the pathway by preventing the translation of intermediary factors. To demonstrate that such an approach could be efficiently used to identify SHR direct targets, we studied the effect of a short-term Dex and Cyc treatment on the translation of GFP in the *shr-2 pSHR::SHR:GR pSCR::GFP* plants. The induction of GFP fluorescence normally observed after 6 h of Dex treatment was entirely inhibited by concurrent treatment with 10 μM Cyc ( Figure 2J and 2K). This indicates that translation was effectively inhibited by the Cyc treatment. Using QRT-PCR, we also observed that *SCR* mRNA is induced in *shr-2 pSHR::SHR:GR* plants after 6 h of Dex treatment even in the presence of Cyc ( Figure S1). This is a first demonstration that SCR is a direct target of SHR and suggests that Cyc effectively inhibits translation without affecting transcription of direct target genes.

Taken together, these results suggests that the SHR:GR fusion protein fully complements the *shr-2* mutant when expressed under the *SHR* endogenous promoter and that the *shr-2 pSHR::SHR:GR* plants can be used to identify the direct targets of SHR.

### A Microarray-Based Meta-Analysis Identifies Direct Endogenous SHR Targets

We next developed a whole-genome meta-analysis approach in order to identify the direct targets of SHR using the inducible SHR protein driven from the SHR native promoter (i.e., the *shr-2 pSHR::SHR:GR* plants). We first performed three independent genome-wide transcriptional analyses after modulation of SHR activity. We obtained transcriptional profiles from root tips of 5-d-old *shr-2 pSHR::SHR:GR* plants treated with Dex and Cyc or with Cyc alone for 6 h using the Affymetrix ATH1 arrays (“direct induction experiment”). As described above, genes differentially expressed between these two treatments are expected to be the direct targets of SHR.

The two other experiments were then chosen in order to minimize the impact of secondary effects and thus the occurrence of false positives. The second experiment, the “loss-of-function” experiment, was designed to identify genes differentially expressed between the *shr* mutant and the wild-type. Since the gross morphological effects of the *shr* mutation become readily apparent only after 5 d, we profiled RNA from the root tips of 5-d-old *shr-2* mutants and wild-type plants. By doing so, we limited the secondary effects of the *shr* mutation on the root transcriptome.

For the third experiment, we used plants expressing a functional SHR:GFP fusion under the control of the regulatory sequences from the *WEREWOLF (WER)* gene (“ectopic expression experiment”). *WER* is transcribed in the epidermis, the lateral root cap (LRC), and epidermal/LRC initials ( Figure 1A) [ 33], which together span a domain that lacks native SHR protein and RNA. Expression of the SHR:GFP fusion has been shown to be sufficient to induce markers of endodermal fate in these cells and to activate asymmetric cell division in the initials [ 34]. To specifically identify the genes activated by SHR in this domain, we used the approach developed by Birnbaum et al. [ 23]. The roots of plants expressing either SHR:GFP ( *pWER::SHR:GFP* plants) or GFP ( *pWER::GFP* plants) in the *WER* domain were dissociated into single cells by enzymatic digestion of their cell walls. The GFP-expressing cells were then sorted with a fluorescence-activated cell sorter, and the RNA population of these cells was analyzed on microarrays. By this approach, we could thus focus specifically on the transcriptome of a single tissue where SHR ectopic expression was proved to activate SHR physiological responses.

The three microarray datasets were then analyzed for differential expression using a mixed-model ANOVA, and the resulting *p*-values were combined in the classical meta-analytic technique of Fisher's inverse χ ^2^ [ 35]. In this process, we selected genes for which at least two experiments showed that SHR regulated their expression level (see Materials and Methods). One of these two experiments had to be the direct induction experiment, since it is the only one that allowed for the inference of direct targets. Importantly, we also verified that the identified genes were not induced by Dex in the absence of the *pSHR::SHR:GR* construct by analyzing on ATH1 microarrays the RNA profiles of roots of 5-d-old wild-type plants treated with Dex or a mock treatment for 6 h (unpublished data).

**Figure 3:**
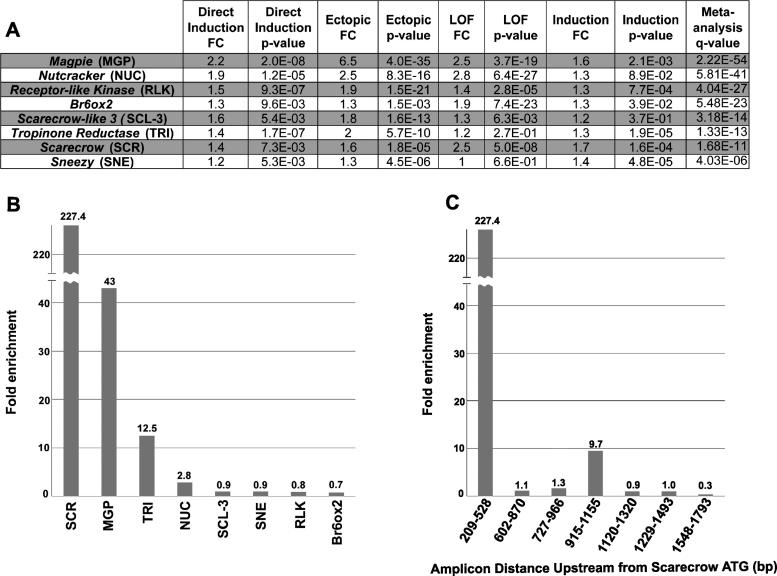
Meta-Analysis Identifies Putative Direct Targets of SHR and Their Promoters Are Bound In Vivo by SHR (A) Identification of SHR direct targets using meta-analysis. The putative direct targets of SHR were identified by combining the results from three independent experiments: the “direct induction,” “ectopic expression,” and “loss-of-function” (LOF) experiments. The fold change (FC) and *p*-values for the direct targets in each experiment are shown. Using a meta-analysis approach, the *p*-values from the three independent approaches were combined, a single meta-analysis *p*-value was calculated for each gene, and the false discovery rate was then estimated by calculating *q*-values (meta-analysis *q*-value). The FC and *p*-value obtained in the “induction experiment” (Ind) are also indicated. (B) Demonstration of SHR binding to the promoter regions of the candidate direct targets using ChIP-QRT-PCR (see Materials and Methods). (C) Tiling of the *SCR* promoter using ChIP-QRT-PCR. Overlapping primers specific to 200- to 350-bp regions along 1.8 kb of the *SCR* promoter were used to identify the regions bound by SHR (see Materials and Methods).

Using this procedure, we identified with high statistical confidence eight candidate direct targets that are positively regulated by SHR ( Figure 3A). Four candidate genes encode putative transcription factors. They are *SCR, SCARECROW-like 3 (SCL-3),* which is another GRAS family member shown to be expressed in the endodermis [ 36], and two closely related C2H2 zinc finger transcription factors that we named *MAGPIE (MGP)* and *NUTCRACKER (NUC).* It is noteworthy that *SCR* was identified as a candidate direct target, given that we already had strong evidence that it was downstream of SHR. The AT5G67280 candidate gene encodes an RLK that belongs to the LRRIII subfamily of RLKs [ 37] and will be referred to as *RLK* in this report. Two candidates encode metabolic enzymes: a tropinone reductase *(TRI),* which may regulate tropane alkaloid synthesis [ 38], and the BR6ox2/Cyp85A2 cytochrome P450 *(BR6ox2),* which is potentially involved in brassinosteroid (BR) biosynthesis [ 39]. The last candidate encodes the F-box protein SNEEZY/SLEEPY 2 *(SNE)* that is thought to play a role in gibberellin signaling [ 40, 41].

### Candidate Direct Targets Are Regulated In Vivo by SHR, and Their Expression Patterns Overlap with the SHR Domain

As one means of confirming that the genes identified by the meta-analysis are direct targets of SHR, we examined binding of SHR in vivo to the promoter region of the candidate genes. We conducted ChIP with a polyclonal antibody to the GFP protein followed by quantitative real-time PCR (ChIP-QRT-PCR) to test for quantitative enrichment of the eight candidate gene promoters ( Figure 3B). Primers to the 5′ upstream sequences of the putative target genes were used for PCR amplification from the immunoprecipitated DNA from wild-type plants compared to immunoprecipitated DNA from transgenic plants expressing the SHR:GFP fusion under control of the native 2.5-kb SHR promoter [ 8]. Using this method, we detected an enrichment of the signal in four of the eight cases ( Figure 3B), with very strong enrichment (227-fold and 43-fold) for two of the targets *(SCR* and *MGP).* For *SCR,* the target with the highest enrichment in the ChIP-QRT-PCR assay, we scanned the promoter with primers and found two peaks of strongest enrichment. One peak was between 209 and 528 bp from the start site of translation and the other was at approximately 1 kb ( Figure 3C). This result indicates that SHR binds to the promoters of four of the direct target candidate genes tested *(SCR*, *MGP, TRI,* and *NUC)* and suggests that there may be more than one binding site for SHR on the *SCR* promoter. The absence of enrichment for the other four candidates is not evidence for a lack of functional binding as it could be related to dilution due to restricted expression domains, lower binding constants, or some combination of these factors. Together with the ability of SHR to activate gene expression in the absence of protein synthesis, this demonstrates that SHR has transcription factor activity and, most important, confirms that at least four of the eight direct targets identified by the meta-analysis are directly bound by SHR in vivo.

**Figure 4:**
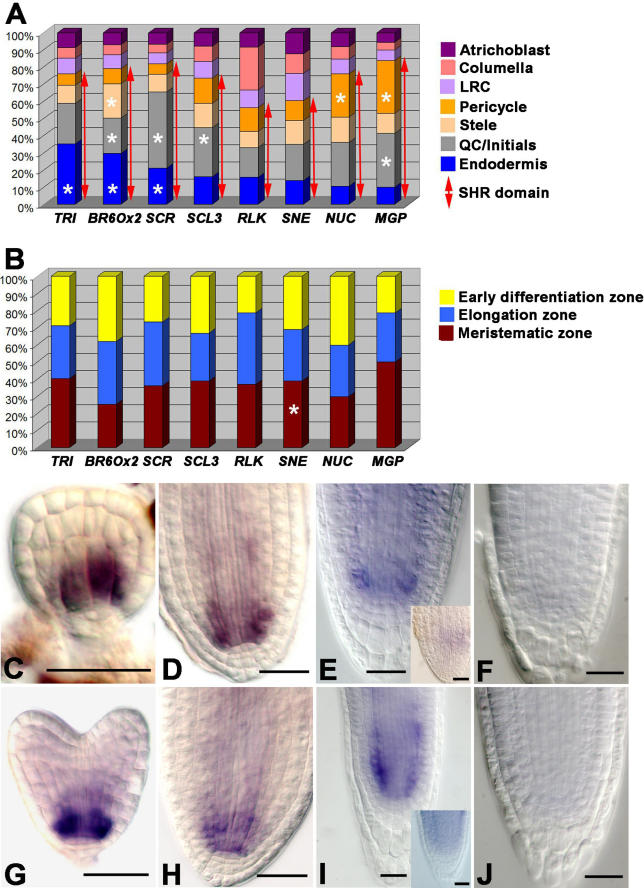
The SHR Direct Targets Are Regulated In Planta by SHR and Are Significantly Enriched in the SHR Domain (A and B) Expression of SHR direct targets in the root using the digital in situ data. (A) Relative expression of the SHR direct targets in the radial zones. (B) Relative expression of the SHR direct targets in the longitudinal root zones. A statistical analysis of the digital in situ data was performed to determine if the SHR direct targets are enriched in tissues in which the SHR protein is present (SHR domain, designated with a red arrow): the quiescent center and initials (QC/initials), endodermis, pericycle, and stele. A similar analysis was performed to determine significant enrichment in a specific longitudinal zone. The asterisk (*) marks the zone in which enrichment was found statistically significant in those analyses. See Materials and Methods for a description of the statistical analysis. (C–F) Whole-mount in situ hybridization of *MGP* in wild-type and *shr-2* embryonic and postembryonic tissues. (C) Wild-type globular embryo. (D) Root of wild-type torpedo embryo. (E) A 2-d-old wild-type root. Inset in (E): A 2-d-old wild-type root hybridized with *MGP* sense probe. (F) A 2-d-old *shr-2* root. (G–J) Whole-mount in situ hybridization of *NUC* in wild-type and *shr-2* embryonic and postembryonic tissues. (G) Wild-type transition embryo. (H) Root of wild-type torpedo embryo. (I) A 2-d-old wild-type root. Inset in (I): A 2-d-old wild-type root hybridized with *MGP* sense probe. (J) A 2-d-old *shr-2* root.

As an alternative approach to confirming our results and a means of gaining insight into the possible function of the putative direct targets of SHR, we asked if their expression patterns were compatible with direct regulation by SHR. We first analyzed their expression patterns in the root using the digital in situ approach [ 26], which measures gene expression among cell and tissue types (radial zones) and along the developmental gradient (longitudinal zones) in the *Arabidopsis* root. To determine specificity of the spatial expression pattern of the putative direct targets, we obtained statistical significance levels for differential expression between the seven previously published radial zone microarray datasets (atrichoblast, columella, LRC, stele, QC/initials, ground tissue, and endodermis: Figure 4A) [ 26, 42] as well as one that we generated from a pericycle-specific enhancer trap line (see Materials and Methods). We performed a similar statistical analysis of differential expression in the longitudinal zone datasets (meristematic, elongation, and early differentiation zones: Figure 4B) [ 26]. The results from this data analysis match precisely the known expression pattern for *SCR* and *SCL-3* [ 7, 36]. It shows that six of the eight putative direct targets of SHR are significantly enriched in a radial cell-type in which SHR protein is present versus all cell-types in which SHR is absent ( Figure 4A). The two exceptions are *SNE* and *RLK,* which both appear to be enriched at low levels in the QC/initials compared to two, but not all three, non-SHR cell-types ( Figure S2). Only *SNE* was significantly enriched in a longitudinal zone, and it is in the meristem ( Figure 4B). Thus, all of the putative direct targets of SHR are either significantly enriched or have some component of their expression in an SHR cell-type, as expected from genes directly regulated by SHR.

The two novel transcription factors *MGP* and *NUC* have not been described previously, and we decided to use RNA in situ hybridization to further analyze their expression pattern in wild-type plants during embryogenesis and in the postembryonic root ( Figure 4C– 4J). *MGP* was first detected in the lower tier of the mid-globular stage embryos in the procambium and the basal cells of the ground meristem ( Figure 4C; embryo stages after [ 43]). This expression was maintained throughout embryogenesis and was progressively restricted to the tip of the embryonic root ( Figure 4D and unpublished data). After the heart stage, *MGP* was most strongly expressed in the vascular stem cells and the basal cells of the ground tissue including the initials. Lower expression levels could also be detected higher in the stele and in the ground tissue. The same expression pattern was seen in the postembryonic root ( Figure 4E). The expression pattern of *NUC* is essentially identical ( Figure 4G– 4I), confirming that *MGP* and *NUC* are expressed in a portion of the SHR expression domain. Moreover, expression of both *MGP* and *NUC* is completely lost in the embryonic and postembryonic root of *shr-2* mutants ( Figure 4F and 4J). SHR is thus necessary to activate and maintain the expression of both genes throughout development, in agreement with a direct regulation of *MGP* and *NUC* by SHR.

In conclusion, we have shown that the promoters of four of the eight putative direct targets we identified were bound in vivo by SHR and that the RNA of the eight putative targets is found in tissues with SHR protein. We have also confirmed by in situ hybridization that SHR is necessary to activate and maintain the expression of two previously uncharacterized transcription factors, *MGP* and *NUC,* with the promoter of these two genes being bound by SHR. These results provide very strong evidence for direct regulation by SHR of four of the eight candidate genes *(SCR, MGP, NUC,* and *TRI).* The expression patterns of the other four targets *(SCL3, SNE, RLK,* and *Br6Ox2)* are consistent with direct regulation by SHR but further analysis will be necessary to confirm direct binding of SHR to their regulatory regions.

### A Role for *SHR* in the Development of the Stele Is Identified

**Figure 5:**
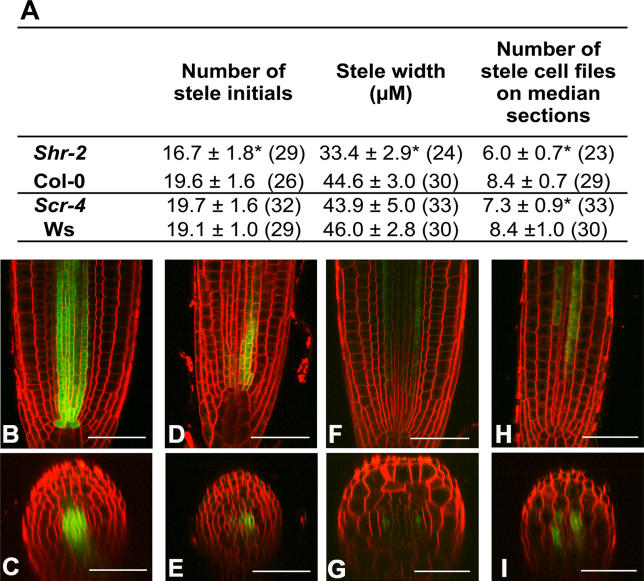
A Mutation in *SHR* Affects the Development of the Stele (A) Cellular parameters of stele development in *shr-2* mutants, *scr-4* mutants, and the corresponding wild-type plants (Col-0 for *shr-2* and Ws for *scr-4*). The number of stele initials, the stele width 100 μm above QC, and the number of stele cell files visible on median longitudinal optical sections in the same region were measured in 4-d-old plants. The results ± standard deviation are indicated, as well as the number of plants analyzed in each case (in between parenthesis). The stars indicate values that are significantly different in the mutant compared to wild-type ( *t*-test: *p* < 0.01). (B–E) Expression of the Q0990 GFP marker in 5-d-old *shr-2* mutants and wild-type plants. (B and D) Longitudinal (B) and transverse (D) optical section of a wild-type root. (C and E) Longitudinal (C) and transverse (E) optical section of a *shr-2* mutant root. (F–I) Expression of the J2094 GFP marker in 5-d-old *shr-2* mutants and wild-type plants. (F and H) Longitudinal (F) and transverse (H) optical section of a wild-type root. (G and I) Longitudinal (G) and transverse (I) optical section of an *shr-2* mutant root. Scale bars: 25 μm.

Several putative direct targets of SHR were found to be expressed in stele tissues *(MGP, NUC,* and *Br6ox2)* which suggested a role for SHR in the stele and prompted us to investigate stele development in *shr* mutants. Previous phenotypic characterization had focused on the dramatic effects on root length and radial patterning [ 6– 8]. We first detected a significant reduction in the number of stele initials in 4-d-old *shr-2* seedlings compared to wild-type plants ( Figure 5A). The *shr-2* mutation also reduces the width of the stele measured at 100 μm above the QC (where the early differentiation of the phloem and the xylem starts in wild-type) and reduces the number of stele cell files visible on median optical sections in the same region ( Figure 5A). The same analysis was performed on the *scr-4* mutant. A significant but much smaller reduction of the number of cell files was observed but no differences were found in the number of stele initials or in the width of the stele 100 μm above the QC. The limited impact of the *scr-4* mutation on stele development suggests that the perturbations observed in *shr-2* mutants are not indirectly caused by the effects of the *shr* mutation on QC activity or ground tissue development. These observations thus suggest that *SHR* plays a direct role in regulating the cell divisions that generate the initials, which in turn determine the number of cell files in the stele.

To determine if *SHR* also plays a role in cell specification in the stele, we analyzed the effect of *SHR* mutation on the expression of two stele-specific markers (Q0990 and J2094). The Q0990 GFP marker is expressed in all the stele initials, with the exception of the pericycle initials ( Figure 5B and 5C). In 19 of 23 *shr-2* plants, Q0990 was only detected in a subset of stele initials or completely absent ( Figure 5D and 5E and unpublished data). Q0990 expression was also generally restricted to the region of the stele above the QC and not observed in the more mature tissues (unpublished data). Moreover, Q0990 was often detected in some pericycle initials in *shr-2* mutants ( Figure 5E and unpublished data). The J2094 marker is specifically expressed in the protophloem in wild-type root tips ( Figure 5F and 5G). In 13 of 23 *shr-2* plants, an expansion of the expression domain of J2094 was observed ( Figure 5H and 5I). These results indicate that the *shr-2* mutation strongly perturbs the specification of the stele initials as well as the patterning of the stele tissues, and notably the phloem and procambium tissues.

Taken together, our data indicate that *SHR* plays a role both in regulating division of the precursors to the stele initials, which probably involves asymmetric cell division as well as a role in regulating the specification of the progeny of these initials. These effects are more subtle than those observed for the ground tissue, and our genomic approach to characterizing the SHR pathway led to their discovery.

### Global Analysis of the SHR Pathway Identifies Transcription Factors and Proteins Involved in Signaling Pathways as Essential Components of the SHR Subnetwork

To further dissect the developmental pathway regulated by SHR, we then conducted a global analysis of the genes regulated by this pathway. Here again, we used a meta-analysis approach to identify the genes indirectly regulated by SHR in several independent experiments. Meta-analysis allowed us to focus on genes primarily affected by the SHR pathway and to minimize experiment-specific secondary effects.

We first generated a new dataset of transcription profiles from the roots of *shr-2 pSHR::SHR:GR* plants treated with Dex or a mock treatment for 6 h (“induction experiment”) without any translational inhibitor. The genes differentially expressed between these two treatments are expected to be those rapidly activated or repressed by the SHR developmental pathway. We then used the meta-analysis approach to combine information from this experiment and from the loss-of-function and the ectopic experiments discussed above. A gene was selected as an indirect target of SHR only if at least two of the experiments indicated that the SHR pathway affected its expression level (see Materials and Methods). Using this approach, we identified 106 genes positively regulated and 389 genes negatively regulated by the SHR developmental pathway ( Table S1). Clustering of the indirect targets using the digital in situ data ( Figure S3; see Materials and Methods) [ 26, 42] indicates that in contrast to the direct targets, the SHR indirect targets are expressed across the entire root and not specifically in the SHR domain.

To understand the functional output of the SHR pathway, we used the Gene Ontology (GO) annotations ( http://www.geneontology.org) of the SHR indirect targets to search for statistically significant overrepresentation of biological processes and molecular functions in the two sets of indirectly regulated genes ( Tables 1 and S2). Of particular interest for understanding how SHR information is relayed along its pathway, this analysis showed a significant overrepresentation in the repressed indirect targets of genes encoding proteins involved in the regulation of transcription (molecular function “transcription factor activity”) and in phosphorylation processes (biological process “protein amino acid phosphorylation” and molecular function “kinase activity” and “serine/threonine kinase activity”). This observation prompted us to look in more detail at SHR indirect targets with predicted transcription factor and kinase activity (see Materials and Methods).

**Table 1:**
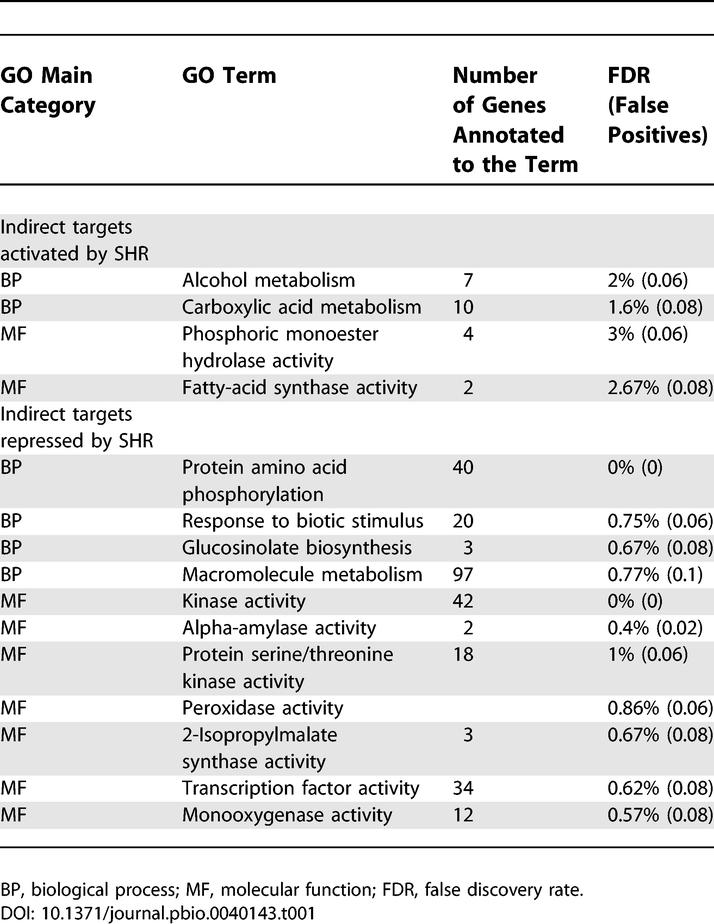
Functional Characterization of SHR Indirect Targets

We identified 46 genes (11.8%) encoding putative transcription factors that are repressed and 10 (9%) that are activated by the SHR pathway ( Table S3), while only 6.3% of the genes expressed in the root are predicted to encode transcription factors (see Materials and Methods). Confirming the results of the GO analysis, this overrepresentation is highly significant for the repressed transcription factors ( *p* < 0.01) but not significant for the activated ones. The digital in situ data show that these transcription factors are expressed throughout the root both radially and longitudinally ( Table S3 and unpublished data). Several transcription factors with known function in development are negatively regulated by the SHR pathway *(PHV, PHB, TNY, ANT,* and *BEL1).* Of particular interest were also several transcription factors encoding regulators of gibberellin and auxin signaling (see below). The SHR pathway thus regulates a large network of transcriptional factors that are broadly expressed in the root.

Of the 389 indirect targets repressed by SHR, we found 37 genes (9.5%) that encode proteins annotated as kinases ( Table S4). Most of these genes (29: 7.45% of the repressed indirect targets) belong to the RLK family [ 44, 45]. The RLKs represent 2.17% of the genes expressed in the root, and the enrichment in the repressed SHR targets is highly significant ( *p* < 0.01). Six of the 106 indirect targets activated by SHR encode proteins annotated as kinases, and three (2.8%) of those encode RLKs. These observations suggest that the SHR pathway also affects different signaling networks using RLKs during root development.

Finally, we focused our attention on genes regulated by SHR and involved in responses to different hormones ( Table S5). We have already described evidence indicating that SHR might directly regulate *SNE* and *BR6ox2,* which suggests a link to gibberellin and BR signaling, respectively. Several genes encoding components of the gibberellin and BR pathway were found downstream of SHR ( Table S5). These observations further support the idea that the SHR pathway interacts with gibberellin and BR signaling. Since auxin has been shown to be an essential player in the root meristem [ 46– 48], we next searched for genes involved in auxin responses. We found 12 genes implicated in auxin-related processes that are repressed by SHR and none among the indirect targets activated by SHR ( Table S5). Most of the repressed genes (seven of 12) are potentially involved in auxin signaling, and the other ones have been implicated in auxin polar transport *(PIN3* and *PIN7)* and auxin homeostasis *(SPS, SUR2,* and *Cyp79B2).* Thus, the SHR pathway appears to also influence auxin signaling, auxin transport, and auxin biosynthesis during root development, but primarily in a negative fashion.

In conclusion, these results show that the SHR pathway regulates the expression of a large number of transcription factors. It also affects the expression of genes involved in signaling such as those encoding RLKs and genes involved in hormonal responses.

## Discussion

SHR is a putative transcription factor of the plant-specific GRAS family of proteins and plays a key role in root development. In this report we have used a microarray-based strategy to identify the effectors of SHR function in root development and provide evidence that SHR directly regulates transcription in planta. Through a meta-analysis approach, we combined statistical information from several independent experiments in which we analyzed transcriptional profiles after modulation of SHR activity. We predicted eight direct targets of SHR. One of the direct targets is *SCR,* which was previously shown to be downstream of SHR. The other seven genes had not been previously implicated in the SHR pathway. We demonstrate that the expression patterns of all of these genes are consistent with direct regulation by SHR, and our data confirm that SHR binds in vivo to the promoter regions of at least four of the putative direct targets. Taken together, our results strongly support a direct regulation of *SCR, MGP, NUC,* and *TRI* by SHR during root development, while further analysis will be necessary to confirm binding of SHR to the regulatory regions of the other four putative direct targets. We also used the meta-analysis approach to perform a global survey of the SHR pathway. Our data reveal the existence of a complex network of transcriptional and signaling events downstream of SHR.

### Meta-Analysis of Microarray Data Provides a Reliable Tool for the Analysis of Developmental Pathways

Consistency of changes in gene expression across several microarray experiments has been widely used to identify genes of potential biological interest, because it has the potential to greatly increase the confidence in the identified genes [ 25, 49]. The meta-analysis approach formalizes the analysis of consistency through the combination of statistical information from multiple studies. Meta-analysis has been used in many fields since the early 20th century, and recently on microarray data, to detect subtle but significant patterns in datasets [ 23, 24, 35, 50– 52]. To our knowledge, this approach has not been previously used to map a developmental pathway in a multicellular organism.

We applied meta-analytic techniques to the identification of the targets of the SHR transcription factor to identify the nodes in the SHR subnetwork. The meta-analysis allowed us to identify the direct targets of SHR using an inducible SHR protein driven from its native promoter *(pSHR::SHR:GR),* while previous studies relied on overexpression under a strong constitutive promoter [ 17, 18, 20, 22]. The statistical tests for significance across these experiments also allowed us to reliably detect targets with low expression in only a few cell types of the root, such as seen for *MGP* and *NUC* ( Figure 4). Our results are thus consistent with the idea that meta-analysis can be used both to ascertain consistency across experiments and to increase the sensitivity of detecting differential expression using microarrays [ 23].

In an effort to identify the binding site for SHR, we used various motif-finding programs, including Motif-Finder, Meme, and Gibbs sampler [ 53, 54] on the four confirmed targets as well as on all eight putative targets (unpublished data). No statistically significant putative binding site with high information content was identified. This may indicate that the training set is too small to predict binding sites with confidence or that the binding site has relatively high degeneracy. It is also possible that heterodimerization of SHR with factors specific to expression subdomains results in binding sites with different position weight matrices. By tiling across the SCR promoter, we found two regions of enriched SHR binding. Interestingly, these coincide with regions that are conserved in phylogenetic footprints across several different *Brassica* species (J. Colinas and PNB, unpublished data). A combination of promoter tiling with ChIP-QRT-PCR, phylogenetic footprinting, and promoter expression analysis should allow us to narrow down the likely binding site(s) of SHR.

The meta-analysis approach was also used to ascertain the indirect targets of SHR, and the genes identified are thus expected with a high level of confidence to be regulated by the SHR pathway. By doing so, we have identified an ensemble of genes constituting the SHR subnetwork and will be able to use this list of candidate genes to explore the next nodes in the SHR subnetwork, through iterative identification of transcription factor direct targets.

### SHR Directly Activates *SCR*

The known functions of SHR are primarily in the QC and endodermis where it regulates stem cell specification and radial patterning, and this function can be explained by the movement of SHR protein into the layer neighboring the stele [ 6– 8]. *SCR* is specifically expressed in all the cells neighboring the stele and was previously shown to act downstream of SHR in the regulation of QC identity and asymmetric cell division [ 6, 9, 11]. We demonstrate in this study that SHR directly regulates the transcription of *SCR* through binding to the chromatin upstream of the gene. Given the cell autonomous requirement of *SCR* in QC specification and asymmetric cell division [ 10, 11], our results show that SHR functions in these two processes partly through direct regulation of the *SCR* gene.

However, it was shown that expression of *SCR* in the QC of *shr* mutants is not sufficient to rescue the division of the initials [ 10]. In addition, when SHR is ectopically expressed under the WER promoter, supernumerary layers are generated through activation of SCR and asymmetric cell division in the epidermis initials [ 34]. In contrast, expression of SCR alone in the *WER* domain does not result in ectopic cell layers (Ji-Young Lee and PNB, unpublished data). SHR function in stem cell specification and asymmetric cell division would thus appear to be mediated by other genes in addition to SCR. The spatial expression of the direct targets we identified suggests that several of them might be implicated in QC and stem cell specification and/or radial patterning in parallel with *SCR. MGP* and *NUC* are homologous genes expressed in the CEI as well as in the first few cells of the endodermal lineage and could thus redundantly regulate the fate of the CEI and radial patterning. The observation that *SCL3* is enriched in the endodermis, QC, and initials [ 36] ( Figure 4) indicates that it could have a similar function to *SCR*. The digital in situ data also suggest that *TRI* is mostly expressed in the endodermis and may play a role in differentiation of the endodermis or in radial patterning.

### Direct Targets in the Stele Reveal a Role for SHR in Vascular Development

Our data revealed that SHR also regulates transcription in the stele and is thus active in its entire domain. In the absence of SHR, two of the confirmed direct targets, *MGP* and *NUC,* fail to express in the stele ( Figure 4), suggesting that their expression in this tissue is entirely dependent upon SHR ( Figure 4). We also observed that mutation of *SHR* results in a diminution in the number of stele initials, perturbs their specification, and affects the differentiation of the phloem ( Figure 5). Our results thus strongly support a role for SHR in cell division and specification within the stele.

A role for SHR in stele development is also suggested by the putative direct target *BR6Ox2.* The encoded enzyme has been implicated in the biosynthesis of the brassinosteroid phytohormones (BRs) [ 39], which are important regulators of vascular development [ 55]. Three other genes involved in regulation of BR response are also indirectly regulated by the SHR pathway ( Table S5). One of these encodes the BR receptor BRL3, which was shown to be involved in the development of the vasculature in synergy with two other genes encoding BR receptors [ 56]. The SHR pathway also regulates the expression of the homologous *Homeodomain Leucine-Zipper III (HD-Zip III)* genes, *PHB* and *PHV.* The HD-Zip III proteins have been shown to be essential regulators of the patterning of vascular tissues in the stem [ 55, 57, 58]. The SHR pathway thus modulates directly and indirectly the expression of essential regulators of vascular development, further supporting a primary role of the SHR pathway in the development of the vascular tissue.

**Figure 6:**
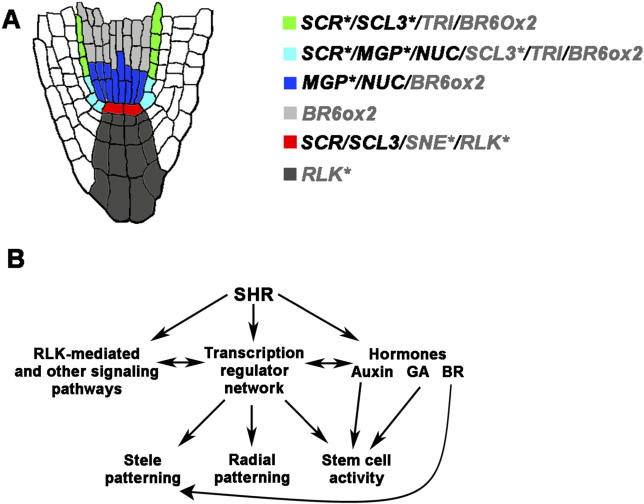
Models for SHR Function in Root Development (A) The interaction of SHR with various coregulators allows for the activation of specific direct targets defining five functional subdomains in the SHR domain: the QC, the early endodermis, the late endodermis, the early stele, and the late stele. The spatial specificity of the different direct targets allows SHR to function in QC specification, CEI asymmetric cell division, early and late endodermal specification, but also early and late stele specification. The gene name was indicated in black or in gray when the expression was inferred from RNA in situ hybridization or digital in situ data, respectively. The stars indicate genes for which binding to the promoter region was demonstrated by ChIP-QRT-PCR. (B) Global model of the SHR developmental pathway. SHR controls root development through the regulation of three interconnected modules: a transcription regulator module, a hormonal module, and a signaling module. The interactions between these three modules determine the developmental output of the pathway on stem cell niche specification, radial patterning, and stele development.

Spatial expression of the confirmed and putative direct targets of SHR defines at least five subdomains in the SHR domain: the QC, the early endodermis, the late endodermis, the early stele, and the late stele ( Figure 6A). The fact that several direct targets of SHR show very different expression patterns indicates that other genes probably act as coregulators with SHR in order to provide this spatial specificity. We thus propose a model where the interaction of SHR with various coregulators allows for the activation of specific direct targets. This defines functional subdomains ( Figure 6A) allowing SHR to function in QC specification, CEI asymmetric cell division, and early and late endodermal specification but also early and late stele specification. The analysis of the function of the different direct targets will allow us to test the validity of this model.

### Output and Global Architecture of the SHR Developmental Pathway

Our global analysis of the genes regulated by the SHR pathway suggests that the SHR pathway acts mostly in a repressive way, since 79% (389 of 495) of the SHR indirect targets are repressed by SHR. Other studies of transcription factor targets have generally found equivalent numbers of indirectly activated and repressed genes in their assays or higher numbers of upregulated genes [ 17, 19, 59, 60].

Our data also begin to reveal the global architecture of the SHR subnetwork. We identified a large number of transcription factors downstream of SHR, with a significant enrichment of transcription factors among the repressed genes ( Tables 1, S2, and S3). This suggests that SHR function in root development is mediated notably through a large network of transcription factors. However, signaling events mediated by posttranscriptional modifications and small molecules such as hormones may also contribute significantly to SHR downstream function. We show that kinases are overrepresented in the set of genes repressed by the SHR pathway and that their number is higher than the genome average in the pool of activated genes as well ( Tables 1, S2, and S4). Seventy-five percent of these genes encode RLKs, and we show that an RLK of unknown function is directly regulated by SHR. This is strong evidence that signaling pathways involving RLKs may play a key role in mediating the SHR response. In addition to the modulation of BR signaling discussed above, the SHR pathway affects the expression of genes involved in both signaling and biosynthesis of two other hormones, gibberellin and auxin ( Table S5). This global effect of the SHR pathway is likely to result in the tuning of hormonal responses and is particularly relevant for stem cell specification in the case of auxin. Auxin has been shown to be essential for positioning the QC [ 2, 47, 48], and the transcriptional regulation of genes involved in auxin responses by the SHR pathway could help to coordinate the action of the two pathways in the specification of the stem cell niche.

Taken together, our results suggest a global model of the SHR pathway where SHR controls root development through the regulation of three interconnected modules: a transcription regulator module, a hormonal module, and a signaling module ( Figure 6B). In this model, the interactions between these three modules determine the developmental output of the pathway on stem cell niche specification, radial patterning, and stele development.

## Materials and Methods

### Plant lines and growth conditions.

Arabidopsis thaliana lines in the Columbia (Col-0), C24, and Wassilewskaya (Ws) ecotypes were used. The *pWER::SHR:GFP* and *pWER::GFP* lines and the *shr-2* mutants are in Col and are described in [ 7, 34, 61]. The *scr-4* mutant is in the Ws ecotype [ 61]. The J2261, J2094, and Q0990 enhancer traps (C24 ecotype) are from the Jim Haselhof collection ( http://www.plantsci.cam.ac.uk/Haseloff/Home.html) and were obtained through TAIR ( http://www.arabidopsis.org). Seeds were surface-sterilized for 8 min in a 0.9% dichloroisocyanuric acid solution. The solution was prepared by adding 440 mg of dichloroisocyanuric acid dissolved in 5 ml of water to 45 ml of ethanol. The seeds were then washed twice in 95% ethanol and left to dry overnight. They were then germinated on plates containing 1× Murashige and Skoog salt mixture, 8.9 mM thiamine, 40.6 mM nicotinic acid, 2.4 mM pyrridoxin, 0.56 mM *myo*-inositol, 1% (w/v) sucrose, and 2.3 mM 2-( *N*-morpholino)ethanesulfonic acid (pH 5.8) in 1% agar. For the plants grown for the microarray experiments, a layer of nylon mesh (Sefar, 03–100/47) was added on top of the agar to facilitate transfer onto treatment media and/or root dissection. For Dex treatments, a 10 mM stock solution was prepared in ethanol and added to the medium at a concentration of 10 μM. For Cyc treatments, a 100 mM stock solution was prepared in DMSO and added to the medium at a concentration of 10 μM. The same amount of ethanol and/or DMSO was added to the control plate as necessary.

### Plasmid construction and plant transformation.

Standard molecular biology techniques were used for all cloning procedures [ 62]. To generate the *pSHR::SHR:GR* fusion gene cassette, a PCR-based megaprimer approach was used to modify the SHR coding sequence and silently eliminate internal SpeI and SacI sites [ 63]. The SHR coding region was amplified from a genomic subclone using two end primers, CAGTCGACTAGTCATATGGATACTCTCTTTAGATTA and AAGAGCTCGGATCCGTTGGCCGCCACGCACT, and an internal primer CTCCGTACGGAGAAGATAGCTC. The resulting PCR fragment contained a SalI/SpeI/NdeI linker sequence at its 5′ end and a BamHI/SacI linker at its 3′ end. This fragment was digested with SalI and SacI and cloned into pBC SK(+) (Stratagene, La Jolla, California, United States) to give pBC-SHR. The 140-bp tobacco etch virus TE was amplified from the plasmid pAVA321 [ 64] with two end primers: CGACTAGTCTCAACACAACATAT and CCCATATGTATCGTTCGTAAATG. This fragment was inserted before the SHR coding sequence in pBC-SHR using the SpeI and NdeI sites incorporated by PCR and generated pBC-TE-SHR. The 0.9-kb GR fragment (corresponding to amino acids 508 to 795) was amplified from the plasmid pBI-ΔGR using the primers GCGGATCCTGGTGGTGAAGCTCGAAAAACAAAG and GTGAGCTCGGGCCCTATTTTTGATGAAACAG. The resulting PCR fragment contained a BamHI site at its 5′ end and an ApaI/SacI linker sequence at its 3′ end, as well as a 12-bp extension at the 5′ end that encoded a short linker sequence (AspProGlyGly). This fragment was inserted after the SHR coding sequence using BamHI and SacI to give pBC-TE-SHR-GR. The TE-SHR-GR fragment was then excised and inserted after the 2.5-kb SHR 5′ sequence in the pBIH plant transformation vector. To generate the *pSCR::GFP* construct, a pBin vector obtained from J. Haselhoff was used to amplify by PCR mGFP5-ER fused to the Nos terminator (NosT). The mGFP5-ER-NosT PCR fragment contained a XbaI/BamHI/XhoI linker at its 5′ end and a KpnI site at its 3′ end. It was inserted in pBlueScript II SK using XbaI and KpnI to generate pSKmGFPL2Nt. The 2-kb region upstream of the ATG of *SCR* was then cloned by PCR from Col-0 genomic DNA. The resulting PCR fragment contained a BamHI linker at its 5′ end and a XhoI at its 3′end. The promoter fragment was inserted as a BamHI-XhoI fragment upstream of mGFP5-NosT in SKmGFPL2Nt. The pSCR::mGFP5-ER-NosT was then inserted in the pCGN1547 binary vector using BamHI and KpnI. The plasmids were then transformed in the *shr-2* mutant or Col-0 wild-type by the floral dip method [ 65].

### Marker analysis and microscopy.

The JIM13 immunolocalization was performed on roots from 5-d-old plants as previously described [ 7]. For confocal microscopy, the roots of 5-d-old plants were stained with 4 μg/ml FM 4–64 (Molecular Probes, Eugene, Oregon, United States) and then washed twice in water before observation with a Zeiss LSM 410 confocal microscope. The number of initials in Col-0 wild-type and *shr-2* mutants was counted on transverse optical sections just above the QC. Stele width was measured 100 μm above the QC using ImageJ ( http://rsb.info.nih.gov/ij, and the number of visible stele files was counted in the same region on median longitudinal optical sections. Whole-mount RNA in situ hybridization on embryos and roots from 2-d-old plants was performed manually using a protocol described by [ 66]. Riboprobes were synthesized using cloned cDNA. The riboprobe spanned the entire coding sequence for *MGP* and from nucleotide 945 to the end of the coding sequence for *NUC*. The images were processed in Photoshop 7.0 (Adobe Systems, San Jose, California, United States) to increase contrast.

### Microarray data acquisition.

The 5-d-old plants were used for all the microarray experiments, and three biological replicates were done for each experiment. For the direct induction, induction and loss-of-function experiments, root tips were dissected at approximately 0.5 cm from the apex and then immediately frozen in liquid nitrogen. For the ectopic experiment, GFP-expressing cells from roots of p *WER::SHR:GFP* and p *WER:::GFP* plants were obtained after protoplasting using fluorescence-activated cell sorting according to [ 26]. The same approach was also used with the enhancer trap line J2661. GFP is specifically expressed in this line in the pericycle in the elongation and differentiation zones of the root (unpublished data). The sorted cells were subsequently frozen before use.

Total RNA was then isolated from the frozen material using the Qiagen RNeasy kit (Valencia, California, United States). Probes for hybridization were then prepared from the total RNA according to standard Affymetrix protocol and hybridized on Affymetrix ATH1 microarray chip.

### Mixed-model analysis.

A mixed-model analysis of variance was performed to identify genes differentially expressed between the various treatments [ 67, 68]. In this approach, a global normalization step was applied to minimize general array-level effects by centering the mean of the log _2_-transformed values to zero for each array [ 68]. Outlier probes with values greater than two standard deviations from the probe-set mean were then removed. Next, a mixed-model ANOVA was applied to the transformed and centered intensity values obtained from the global normalization step. This gene model, which is based on that developed by Chu et al. [ 68], can be formalized as:



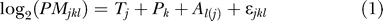



where the PM variable refers to the output of the global normalization procedure for each gene, as described above. The symbols *T, P,* and *A* represent treatment, probe, and array effects, respectively. The array effect *A _l(j)_* is assumed to be a normally distributed random effect [ 68]. A standard error term *ɛ _jkl_* was also applied to this model. In addition, the indices *j, k,* and *l* represent the *jth* treatment, on the *kth* probe, and on the *lth* replicate [ 68]. The output of this model is the mean expression value for every gene, based on the global model, as well as a *p*-value from the gene model for the probability of falsely rejecting the null hypothesis of no-differential expression (α = .05). The global and gene models were run on a Linux server with the statistical software SAS (version 8.2).

### Meta-analysis and selection of the candidate targets.

The meta-analysis was done using Fisher's inverse χ ^2^ method [ 35]. An S statistic was calculated for each gene from the three original *p*-values from each test of differential expression:



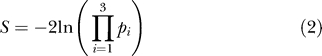



A χ ^2^ test with 6 degrees of freedom was then used to calculate a *p*-value for the likelihood that a gene could be falsely considered to be differentially expressed when all three experiments were combined, given the expected distribution [ 24, 35]. The *p*-values were then adjusted for multiple-hypothesis testing using the q-value false discover rate procedure [ 69]. The predicted false-positive rate (PFR) was set to less than or equal to 1 to reduce the reporting of false positives in the final candidate gene list. All computation for the meta-analysis was done using the statistical analysis software R (version 1.9.0 alpha), which includes a module for performing the q-value calculation. All default parameters in the q-value module were used.

For the direct targets, the meta-analysis yielded 3,866 genes that met the conservative criteria of a q-value threshold <0.0001 (PFR = 0.38). However, the multiplicative nature of the Fisher combination may allow a single low *p*-value from any one of the three microarray experiments to overwhelm the other two in the meta-analysis [ 50]. Thus, we added an additional requirement that candidates must have a statistically significant differential expression in at least two experiments ( *p* < 0.01). One of these experiments had to be the direct induction experiment, since it is the only one that demonstrates direct regulation. We also required the candidate direct targets to be identified as an indirect target in order to minimize artifacts linked to the toxicity of Cyc and to further increase our confidence in the putative direct targets.

For the indirect targets, 3,745 genes met the conservative criteria of a q-value threshold <0.0001 (PFR = 0.37). A gene was then selected as a candidate if the *p*-values for differential expression were below 0.01 in at least two of the three experiments. The direct targets were then removed from the list of candidate indirect targets before further analysis.

### Statistical analysis of the digital in situ data.

The significance of differential expression between all pairwise combinations of tissue-specific transcriptional profiles was obtained using the mixed-model analysis described above. For each pairwise comparison, the predicted PFR was estimated by calculating q-values and the q-value threshold was set to a value between 0.75 and 1.5 (see Table S6 for the PFR used in each comparison). The different tissues compared were (1) SHR cell-types (i.e., stele, QC/initials, endodermis, pericycle) and (2) non-SHR cell-types (i.e., columella, LRC, and atrichoblast). A gene was considered to be enriched in a SHR cell-type if it had statistically significant enrichment in the tissue versus all the non-SHR cell-types.

### Clustering.

The radial and longitudinal expression values for the indirectly induced and indirectly repressed candidates were separately clustered by principal component analysis using the Cluster program [ 70] after log transformation of the original absolute expression values from the mixed model. The number of clusters present in each of these four datasets was then used to set the *y*-dimension of the self-organized map (SOM) corresponding to that dataset, also using Cluster. The SOMs with the principal component analysis−determined *y*-dimension were generated with 100,000 iterations of gene expression values for each radial or longitudinal zone. The program TreeView (version 1.6) was used to visualize the SOM.

### GO analysis and biological theme representation analysis.

The GO analysis was performed using the Web-based generic GO Term Finder, which uses the hypergeometric distribution to look for significant GO terms ( http://go.princeton.edu/cgi-bin/GOTermFinder; [ 71]). To limit redundancy in the same GO main category (Biological Process, Molecular Function), we omitted parent categories synonymous with a child category when the gene content of the parent and child category overlapped by more than two thirds. To identify genes with predicted kinase or transcription factor activity and genes involved in hormone response (auxin, gibberellin, BR), we used Microsoft Access to search the annotations of the SHR targets with the corresponding keywords. For the transcription factors we also used several lists of transcription factors obtained from the AGRIS ( http://arabidopsis.med.ohio-state.edu) [ 72] and DATF ( http://datf.cbi.pku.edu.cn) [ 73] databases and from the Sheen lab Web site ( http://genetics.mgh.harvard.edu/sheenweb). To identify the RLK in the SHR targets, we used the list established by [ 44]. The lists of genes obtained were then manually curated. Statistical significance of enrichment over the total number of transcription factors and RLKs expressed in the root was tested using the hypergeometric distribution. To identify genes expressed in the root, we used the expression values from the mixed-model analysis and proceeded as described in [ 26]. The threshold was set at 1 for the mixed-model analysis values, which is equivalent to the threshold of 75 used by [ 26].

### Chromatin immunoprecipitation followed by gene-specific quantitative real-time PCR (ChIP-QRT-PCR).

ChIP was conducted as in Leibfried et al. [ 74] on roots of 5-d-old wild-type seedlings and 5-d-old seedlings expressing the *SHR:GFP* transgene expressed under the control of the SHR 2.5-kb promoter [ 8]. Immunoprecipitation was done using a rabbit polyclonal antibody to GFP (ab290; Abcam Ltd., Cambridge, United Kingdom). Enrichment of putative target promoter-region DNA was determined using QRT-PCR (as in Leibfried et al. [ 74]). Precipitates from wild-type and SHR:GFP were compared after normalization to input DNA (i.e., sonicated, pre-ChIP DNA). A PCR efficiency of 1.8-fold amplifications per cycle was assumed, and sequences from *Heat-Shock Factor1 (HSF1)* were used to normalize the results between samples (Leibfried et al. [ 74]). Each experiment was done in triplicate with the mean fold enrichments shown in Figure 3B. The following primers were used: SCR (At3g54220): pSCR-F (5′ AGAAACGAAATGGATCGGCAAACG 3′) and pSCR-R (5′ ATTTGGAAGGATGTGGGTTGGAGA 3′); SCL3 (At1g50420): pSCL3-F (5′ TTTTGGGAGTGAGAGGGTTC 3′) and pSCL3-R (5′ AGATGGATGGGATTGGAAAA 3′); MGP (At1g03840): pMGP-F (5′ TCTTTGACCGCCTCAATTTACGGT 3′) and pMGP-R (5′ TTGATCTGTAAGAACTGTCGCAGC 3′); RLK (At5g67280): pRLK-F (5′ GCGTAATCTCACGTCACAATTTCCG 3′) and pRLK-R (5′ TGCTGACGTCGCTTTGTCGTTT 3′); SNEEZY (at5g48170): pSNE-F (5′ TTCTGAAAGTGGGCAAGGAC 3′) and pSNE-R (5′ AAGCGTGGAGGAGACAAAGA 3′); TRI (At2g29330): pTRI-F (5′ TTGGCCGTGTTGGAGAGC 3′) and pTRI-R (5′ GTTGGCGTAGCGGGTGTAA 3′); NUC (At5g44160): pNUC-F (5′ CTCGCTTCGAATTTGCAAGGCTAT 3′) and pNUC-R (5′ GCACCCTATGTTTGCAGTTTCACT 3′); Br6ox2(At3g30180): pCYT-F (5′ CGCGATCTCCACCGTAAT 3′) and pCYT-R (5′ CGAAAATAAGTAAAGGCGAGAT 3′); HSF1 (At4g17750): G-4680 (5′ GCTATCCACAGGTTAGATAAAGGAG 3′) and G-4681 (5′ GAGAAAGATTGTGTGAGAATGAAA 3′).

Tiling and fold enrichment along the SCR promoter was done using the ChIP-QRT-PCR protocol as described above. The following sets of adjacent or overlapping primers specific to 200- to 350-bp regions along 1.8 kb of the *SCR* promoter were used, in ascending order upstream from the SCR ATG: pSCR-F (5′ AGAAACGAAATGGATCGGCAAACG 3′) and pSCR-R (5′ ATTTGGAAGGATGTGGGTTGGAGA 3′); pSCR-F2 (5′ CTAGTGGTGCAACCTGCTGA 3′); pSCR-R2 (5′ TTCGTGGAACCGGTACAATA 3′); pSCR-F3 (5′ AGTTGGTGCCCCATCTTAGT 3′); pSCR-R3 (5′ TCATTATGTGAAATGAATGGGTTT 3′); pSCR-F4 (5′ CGTCTTGTCCAATTCCTCTCA 3′) pSCR-R4 (5′ TCAAAGTGTGGTACGATGTGC 3′); pSCR-F5 (5′ AGAAACAAAAGGGAAAAGATGAGG 3′) pSCR-R5 (5′ AAAGGCATTTTACTTGAGAGGAA 3′); pSCR-F6 (5′ ATCGTAGAAAGCGTGGATGG 3′) pSCR-R6 (5′ CCAACTGTGAAACCCCAGTT 3′); pSCR-F7 (5′ TGGATAAATTTTGGGAAAATCC 3′) pSCR-R7 (5′ AACACAAACACACGGCTCAA 3′).

## Supporting Information

Figure S1 Time-Course after Induction of SHR Demonstrates an Increase in SCR Transcript after 6 h Quantitative real-time PCR was used to measure the level of SCR transcript when *shr-2 pSHR::SHR:GR* plants were treated with a mock treatment, 10 μM Dex, 10 μM Dex plus 10 μM Cyc, or 10 μM Cyc.
(260 KB PDF)

Figure S2 The Statistical Analysis of the Digital In Situ Data Predicts an Enrichment of SHR Direct Targets in the Quiescent Center A statistical analysis of the digital in situ data was performed to determine if the SHR direct targets are enriched in the quiescent center (QC) as compared to tissues where SHR is absent: LRC, columella, and atrichoblast. The stars indicate significant enrichment. See Materials and Methods for a description of the methods used in this analysis.
(9.1 MB TIF)

Figure S3 Indirectly Induced SHR Targets Are Broadly Expressed throughout the Root, and Indirectly Repressed SHR Targets Are Enriched in the Differentiation Zone (A) Self-organizing map clusters of the SHR indirect targets in the root radial zones. High expression of clusters in a tissue are designated in blue, moderate expression is in black, and low-to-absent expression is in red. See Materials and Methods for details on how clusters were obtained. (B) Self-organizing map clusters of the SHR indirect targets in the root longitudinal zones, which includes the meristematic zone (MZ), elongation zone (EZ), and young differentiation zone (YDZ), as in [ 26]. Expression levels are designated as in (A). (C) Statistical analysis of the digital in situ data for SHR indirect targets. The number of indirect targets enriched in the different cell types of the SHR domain and in the longitudinal zones is indicated. The total number of genes expressed in the root and enriched in the different zone is also indicated (total genes). See Materials and Methods for a description of the statistical analysis. QC, quiescent center.
(579 KB PDF)

Table S1 SHR Indirect Targets (533 KB DOC)

Table S2 GO Analysis of SHR Indirect Targets (53 KB DOC)

Table S3 SHR Indirect Targets Encoding Transcription Factors (122 KB DOC)

Table S4 SHR Indirect Targets with Kinase Activity (83 KB DOC)

Table S5 SHR Indirect Targets Involved in Hormone-Related Processes (93 KB DOC)

Table S6 Statistical Analysis of the Radial Zone Digital In Situ Data (44 KB DOC)

### Accession Numbers

The Arabidopsis Genome Initiative ( http://www.arabidopsis.org) accession numbers for the genes and gene products discussed in this paper are *WEREWOLF (WER)* (AT5G14750), SHORT-ROOT (SHR) (AT4G37650), *SCARECROW (SCR)* (AT3G54220), SCARECROW-like 3 *(SCL-3)* (AT1G50420), *MAGPIE (MGP)* (AT1G03840), NUTCRACKER *(NUC)* (AT5G44160), tropinone reductase *(TRI)* (AT2G29330), and BR6ox2/Cyp85A2 cytochrome P450 *(BR6ox2)* (AT3G30180).

## Abbreviations

BR: brassinosteroid
CEI: cortex/endodermis initial
ChIP: chromatin immunoprecipitation
Cyc: cycloheximide
Dex: dexamethasone
GFP: green fluorescent protein
GO: Gene Ontology
GR: glucocorticoid receptor
LRC: lateral root cap
PFR: predicted false-positive rate
QC: quiescent center
QRT: quantitative real-time
RLK: receptor-like kinase
SHR: SHORT-ROOT
SOM: self-organized map
TE: translational enhancer element
TRI: tropinone reductase
