# Correction: Whole-Genome Analysis of the SHORT-ROOT Developmental Pathway in Arabidopsis

**DOI:** 10.1371/journal.pbio.0040249

**Published:** 2006-05-02

**Authors:** Mitchell P Levesque, Teva Vernoux, Wolfgang Busch, Hongchang Cui, Jean Y Wang, Ikram Blilou, Hala Hassan, Keiji Nakajima, Noritaka Matsumoto, Jan U Lohmann, Ben Scheres, Philip N Benfey

## Abstract

Meta-analysis to combine the results of several microarray experiments reveals a new function for the transcription factor SHORT-ROOT in the development of vascular tissue.

In *PLoS Biology*, volume 4, issue 5: DOI: 10.1371/journal.pbio.0040143

There has been a switch in panels in the legend for Figure 5. Panels C and D were inadvertently switched as are panels G and H.

“(B and D) Longitudinal (B) and transverse (D) optical section of a wild-type root. (C and E) Longitudinal (C) and transverse (E) optical section of a *shr-2* mutant root” should be “(B and C) Longitudinal (B) and transverse (C) optical section of a wild-type root. (D and E) Longitudinal (D) and transverse (E) optical section of a *shr-2* mutant root.”

“(F and H) Longitudinal (F) and transverse (H) optical section of a wild-type root. (G and I) Longitudinal (G) and transverse (I) optical section of a *shr-2* mutant root” should be “(F and G) Longitudinal (F) and transverse (G) optical section of a wild-type root. (H and I) Longitudinal (H) and transverse (I) optical section of a *shr-2* mutant root.”

