# Supplementary material for: Whole-Genome Analysis of the SHORT-ROOT Developmental Pathway in Arabidopsis
Source: PLoS Biol. 2006 May 2;4(5):e143. doi: 10.1371/journal.pbio.0040143 (PMC1450008; doi:10.1371/journal.pbio.0040143)
Supplement: Supplementary file 1 [file pbio.0040143.sg001.pdf]

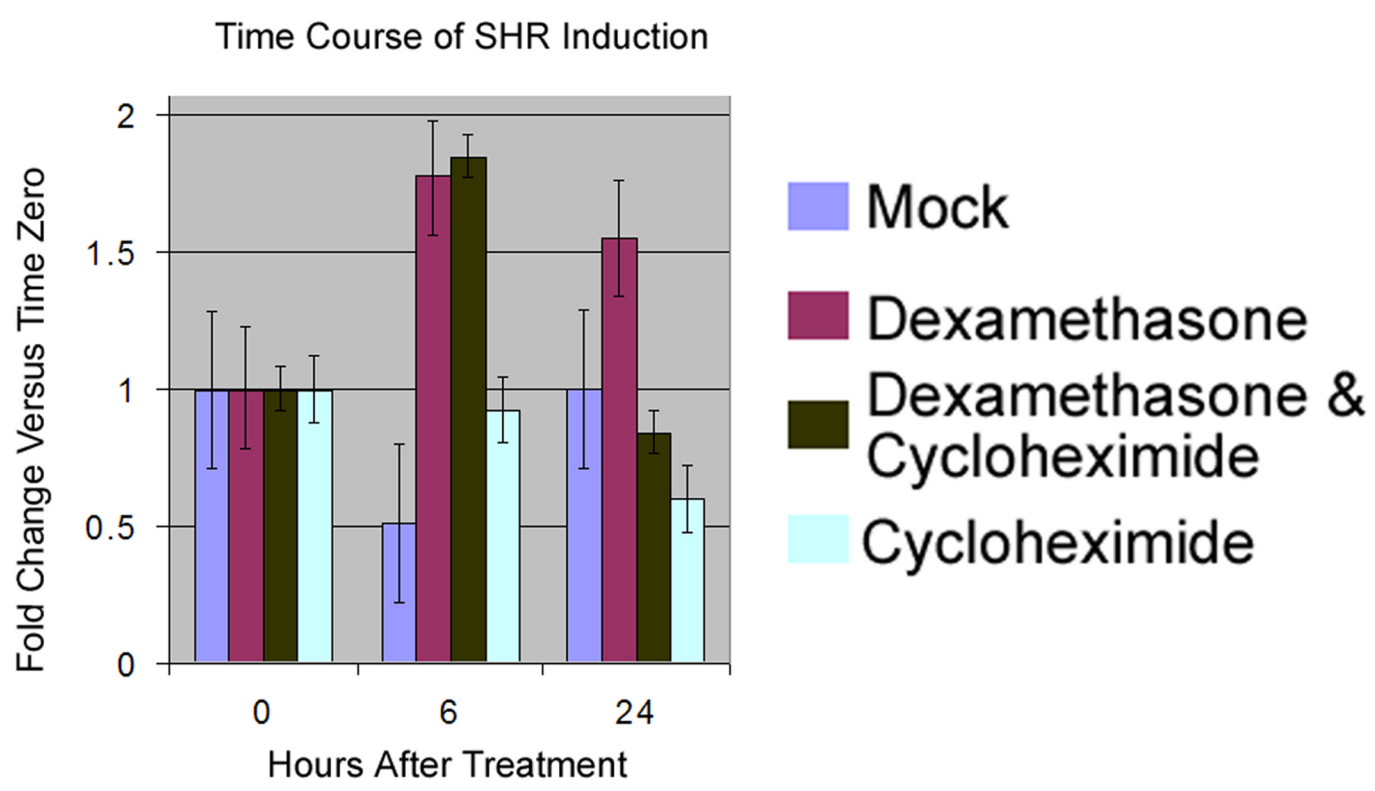
Time Course of SHR Induction
Fold Change Versus Time Zero
2
1.5
1
0.5
0
0
6
24
Hours After Treatment
Mock
Dexamethasone
Dexamethasone &
Cycloheximide
Cycloheximide
