# Supplementary material for: Whole-Genome Analysis of the SHORT-ROOT Developmental Pathway in Arabidopsis
Source: PLoS Biol. 2006 May 2;4(5):e143. doi: 10.1371/journal.pbio.0040143 (PMC1450008; doi:10.1371/journal.pbio.0040143)
Supplement: Supplementary file 2 [file pbio.0040143.sg003.pdf]

A

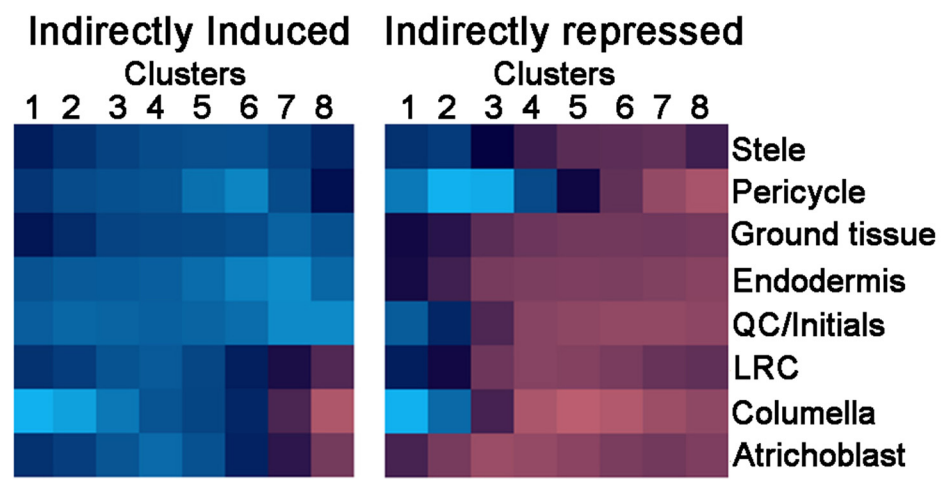

B

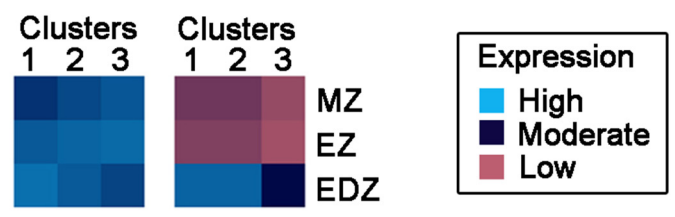

C

	Total Genes	106 Indirectly Induced	389 Indirectly Repressed
**QC/Initials**	582 (4.1%)	4 (4%)	10 (3%)
**Endodermis**	403 (2.8%)	8 (8%)	6 (2%)
**Pericycle**	1077 (7.6%)	5 (5%)	70 (18%)
**Stele**	369 (2.6%)	2 (2%)	33 (8%)
**Union of genes enriched in radial SHR cell-types**	1883 (13.3%)	14 (13%)	91 (23%)
**Meristematic**	1140 (8%)	1 (1%)	18 (5%)
**Elongation**	172 (1.2%)	6 (6%)	0
**Young Differentiation**	1226 (8.5%)	7 (7%)	94 (24%)
