# Supplementary material for: Whole-Genome Analysis of the SHORT-ROOT Developmental Pathway in Arabidopsis
Source: PLoS Biol. 2006 May 2;4(5):e143. doi: 10.1371/journal.pbio.0040143 (PMC1450008; doi:10.1371/journal.pbio.0040143)
Supplement: Supplementary file 3 [file pbio.0040143.st001.doc]

**Table S1. SHR indirect targets**

| **Gene locus** | **Name** | **Tissue/Zone in which the gene is enriched** |
| --- | --- | --- |
| **Activated by the SHR pathway** | |  |
| **AT1G03180** |  |  |
| **AT1G06070** |  |  |
| **AT1G06210** |  |  |
| **AT1G10150** |  |  |
| **AT1G16370** |  | Pericycle |
| **AT1G17160** |  | Elongation Zone |
| **AT1G17550** |  |  |
| **AT1G19180** |  |  |
| **AT1G20270** |  |  |
| **AT1G24360** |  |  |
| **AT1G27460** | ***NPGR1*** | QC/Initials |
| **AT1G34430** |  |  |
| **AT1G35510** |  |  |
| **AT1G43160** |  |  |
| **AT1G51540** |  | Elongation Zone |
| **AT1G53885** |  |  |
| **AT1G56580** |  |  |
| **AT1G64170** |  |  |
| **AT1G65310** |  | Early Differentiation Zone |
| **AT1G72480** |  |  |
| **AT1G72800** |  |  |
| **AT1G72850** |  | QC/Initials, Meristematic Zone |
| **AT1G74960** | ***FAB1/KAS2*** |  |
| **AT1G76550** |  |  |
| **AT1G76590** |  |  |
| **AT1G79610** |  |  |
| **AT1G80670** |  |  |
| **AT2G24850** | ***TAT3*** |  |
| **AT2G29050** |  |  |
| **AT2G29390** | ***SMO2-1*** | Elongation Zone |
| **AT2G29440** |  |  |
| **AT2G36950** |  |  |
| **AT2G39200** |  |  |
| **AT2G40000** |  |  |
| **AT2G42040** |  |  |
| **AT2G44130** |  |  |
| **AT2G47630** |  | Endodermis |
| **AT2G48030** |  | Early Differentiation Zone |
| **AT3G04520** |  | QC/Initials, Endodermis |
| **AT3G11210** |  |  |
| **AT3G11930** |  | Endodermis, Stele, Pericycle, Early Differentiation Zone |
| **AT3G13620** |  |  |
| **AT3G14720** |  |  |
| **AT3G15690** |  |  |
| **AT3G20780** | ***TOP6B/BIN3/HYP6*** |  |
| **AT3G22231** |  |  |
| **AT3G26744** | ***ICE1*** |  |
| **AT3G28290** |  |  |
| **AT3G45430** |  |  |
| **AT3G45970** |  |  |
| **AT3G54400** |  |  |
| **AT3G59140** |  | Elongation Zone |
| **AT4G01410** |  |  |
| **AT4G16120** | ***COBL7/AtSEB1*** |  |
| **AT4G16155** | ***PTLPT2*** |  |
| **AT4G17720** |  |  |
| **AT4G21830** |  |  |
| **AT4G25260** |  | Elongation Zone |
| **AT4G27680** |  |  |
| **AT4G30280** | ***AtXTH18*** | Early Differentiation Zone |
| **AT4G30500** |  |  |
| **AT4G31750** |  |  |
| **AT4G36250** |  |  |
| **AT4G37400** |  |  |
| **AT4G37610** | ***AtBT5*** |  |
| **AT5G01320** |  |  |
| **AT5G01490** |  |  |
| **AT5G02170** |  |  |
| **AT5G02230** |  |  |
| **AT5G04120** |  |  |
| **AT5G08415** |  |  |
| **AT5G09760** |  | Pericycle |
| **AT5G11920** |  |  |
| **AT5G12970** |  |  |
| **AT5G14420** |  |  |
| **AT5G15150** | ***AtHB3/HAT7*** | Endodermis |
| **AT5G16270** |  |  |
| **AT5G17210** |  | Pericycle |
| **AT5G17310** |  |  |
| **AT5G19110** |  |  |
| **AT5G22580** |  |  |
| **AT5G24380** | ***YSL2*** | Endodermis, Pericycle |
| **AT5G24900** | ***CYP714A2*** | QC/Initials |
| **AT5G25770** |  | Endodermis, Early Differentiation Zone |
| **AT5G27670** |  |  |
| **AT5G36890** |  |  |
| **AT5G37500** | ***GORK*** |  |
| **AT5G40610** |  |  |
| **AT5G42580** |  |  |
| **AT5G42700** |  |  |
| **AT5G46910** |  |  |
| **AT5G47050** |  |  |
| **AT5G48450** |  |  |
| **AT5G48900** |  |  |
| **AT5G53090** |  |  |
| **AT5G55180** |  |  |
| **AT5G57350** | ***AHA3*** | Early Differentiation Zone |
| **AT5G57620** |  |  |
| **AT5G58300** |  | Endodermis, Stele |
| **AT5G58600** |  |  |
| **AT5G59480** |  | Elongation Zone |
| **AT5G62420** |  |  |
| **AT5G62500** |  |  |
| **AT5G62580** |  |  |
| **AT5G63410** |  | Endodermis |
| **AT5G67020** |  | Early Differentiation Zone |
|  |  |  |
| **Repressed by the SHR pathways** | |  |
| **AT1G01170** |  |  |
| **AT1G01640** |  |  |
| **AT1G02220** |  |  |
| **AT1G02475** |  |  |
| **AT1G02950** |  |  |
| **AT1G03310** |  |  |
| **AT1G03820** |  | Stele |
| **AT1G04550** | ***IAA12/BDL*** | Stele, Pericycle |
| **AT1G05190** |  | Meristematic Zone |
| **AT1G05250** |  | Early Differentiation Zone |
| **AT1G05470** | ***CVP2*** |  |
| **AT1G05700** |  |  |
| **AT1G05760** | ***RTM1*** |  |
| **AT1G07090** |  | Pericycle |
| **AT1G07130** |  |  |
| **AT1G07700** |  |  |
| **AT1G08230** |  | Pericycle, Early Differentiation Zone |
| **AT1G10750** |  |  |
| **AT1G11330** |  | Pericycle |
| **AT1G11920** |  |  |
| **AT1G12010** |  |  |
| **AT1G12080** | ***KEULE*** | Stele, Pericycle |
| **AT1G12200** |  | Pericycle |
| **AT1G12560** |  | Early Differentiation Zone |
| **AT1G13670** |  |  |
| **AT1G13920** |  |  |
| **AT1G14070** |  |  |
| **AT1G14720** |  |  |
| **AT1G16410** | ***SPS/BUS/CYP79F1*** | |
| **AT1G17100** |  | Pericycle |
| **AT1G19650** |  |  |
| **AT1G19850** | ***MP*** | Meristematic Zone |
| **AT1G19960** |  |  |
| **AT1G20650** |  | Pericycle, Early Differentiation Zone |
| **AT1G21590** |  | Pericycle |
| **AT1G22030** |  |  |
| **AT1G22500** |  | Early Differentiation Zone |
| **AT1G22885** |  | Stele, Pericycle |
| **AT1G23080** | ***PIN7*** |  |
| **AT1G23140** |  | Early Differentiation Zone |
| **AT1G23160** |  | Meristematic Zone |
| **AT1G24735** |  |  |
| **AT1G30490** | ***PHV/ATHB-9*** |  |
| **AT1G30650** | ***WRKY14*** |  |
| **AT1G30840** |  | Pericycle |
| **AT1G30870** |  | Early Differentiation Zone |
| **AT1G31320** |  |  |
| **AT1G33260** |  |  |
| **AT1G33700** |  | Stele, Pericycle, Early Differentiation Zone |
| **AT1G33970** |  |  |
| **AT1G34040** |  | Early Differentiation Zone |
| **AT1G44050** |  | Early Differentiation Zone |
| **AT1G48930** |  | Early Differentiation Zone |
| **AT1G49160** |  |  |
| **AT1G49230** |  |  |
| **AT1G49310** |  | Early Differentiation Zone |
| **AT1G49390** |  | QC/Initials, Meristematic Zone |
| **AT1G49860** |  |  |
| **AT1G51170** |  | Pericycle |
| **AT1G51830** |  |  |
| **AT1G51860** |  | Early Differentiation Zone |
| **AT1G51940** |  |  |
| **AT1G52310** |  |  |
| **AT1G52880** |  |  |
| **AT1G54740** |  |  |
| **AT1G55740** |  | Stele, Pericycle, Early Differentiation Zone |
| **AT1G55760** |  |  |
| **AT1G57560** |  | Early Differentiation Zone |
| **AT1G59124** |  |  |
| **AT1G59850** |  | Early Differentiation Zone |
| **AT1G61860** |  | Early Differentiation Zone |
| **AT1G61930** |  | Early Differentiation Zone |
| **AT1G62320** |  | Early Differentiation Zone |
| **AT1G62510** |  | Early Differentiation Zone |
| **AT1G62980** | ***AtEXP18*** | Early Differentiation Zone |
| **AT1G63310** |  |  |
| **AT1G64160** |  |  |
| **AT1G64405** |  |  |
| **AT1G64630** |  |  |
| **AT1G64690** |  |  |
| **AT1G64780** |  |  |
| **AT1G64970** | ***γ-TMT/VTE4*** |  |
| **AT1G65500** |  |  |
| **AT1G66350** | ***RGL1*** | Meristematic Zone |
| **AT1G66440** |  |  |
| **AT1G67500** | ***AtREV3*** | Meristematic Zone |
| **AT1G67520** |  |  |
| **AT1G67830** |  | QC/Initials |
| **AT1G68810** |  | Stele |
| **AT1G68880** |  | Pericycle |
| **AT1G69440** | ***AGO7/ZIPPY*** |  |
| **AT1G70460** |  | Early Differentiation Zone |
| **AT1G70470** |  |  |
| **AT1G70550** |  |  |
| **AT1G70880** |  |  |
| **AT1G70940** | ***PIN3*** |  |
| **AT1G71695** |  | Stele |
| **AT1G71880** | ***SUC1*** | Endodermis, Stele, Pericycle, Early Differentiation Zone |
| **AT1G72220** |  | Early Differentiation Zone |
| **AT1G73260** |  |  |
| **AT1G73270** |  |  |
| **AT1G73600** |  |  |
| **AT1G73780** |  | Stele |
| **AT1G74440** |  |  |
| **AT1G74660** |  | Pericycle, Early Differentiation Zone |
| **AT1G75410** |  | Early Differentiation Zone |
| **AT1G75500** |  | Stele, Pericycle |
| **AT1G76040** |  | Endodermis, Pericycle |
| **AT1G76930** |  |  |
| **AT1G77020** |  | Stele, Pericycle |
| **AT1G77280** |  |  |
| **AT1G77380** |  |  |
| **AT1G77920** |  | Early Differentiation Zone |
| **AT1G78020** |  |  |
| **AT1G78120** |  |  |
| **AT1G79250** |  |  |
| **AT1G79580** |  | Meristematic Zone |
| **AT2G01900** |  |  |
| **AT2G02100** |  | Stele, Pericycle |
| **AT2G02130** |  | Endodermis, Stele, Pericycle, Early Differentiation Zone |
| **AT2G03720** |  | Early Differentiation Zone |
| **AT2G05620** | ***PGR5*** |  |
| **AT2G13820** |  | Endodermis, Stele, Pericycle |
| **AT2G16700** |  |  |
| **AT2G18140** |  | Endodermis |
| **AT2G18350** |  |  |
| **AT2G18450** |  | Early Differentiation Zone |
| **AT2G19990** |  |  |
| **AT2G21020** |  | Early Differentiation Zone |
| **AT2G21550** |  |  |
| **AT2G23030** |  |  |
| **AT2G23590** |  | Early Differentiation Zone |
| **AT2G24150** |  | Stele |
| **AT2G24280** |  |  |
| **AT2G25000** |  | Early Differentiation Zone |
| **AT2G25090** |  |  |
| **AT2G25240** |  | Early Differentiation Zone |
| **AT2G26410** |  |  |
| **AT2G26440** |  | QC/Initials |
| **AT2G26820** |  |  |
| **AT2G27140** |  |  |
| **AT2G28260** |  |  |
| **AT2G28350** | ***ARF10*** |  |
| **AT2G28720** |  |  |
| **AT2G29320** |  |  |
| **AT2G29660** |  | Pericycle |
| **AT2G29730** |  | Pericycle |
| **AT2G30150** |  | Stele, Pericycle |
| **AT2G30840** |  |  |
| **AT2G31160** |  |  |
| **AT2G31380** |  |  |
| **AT2G32430** |  |  |
| **AT2G33770** |  | Pericycle, Early Differentiation Zone |
| **AT2G33850** |  |  |
| **AT2G34710** | ***PHB*** | Meristematic Zone |
| **AT2G34790** |  |  |
| **AT2G35155** |  |  |
| **AT2G36090** |  |  |
| **AT2G36210** |  |  |
| **AT2G36870** |  | Pericycle |
| **AT2G37820** |  | Early Differentiation Zone |
| **AT2G39360** |  | Early Differentiation Zone |
| **AT2G39370** |  | Pericycle |
| **AT2G39690** |  | Early Differentiation Zone |
| **AT2G40150** |  | Pericycle, Early Differentiation Zone |
| **AT2G40480** |  | Pericycle |
| **AT2G40530** |  |  |
| **AT2G41300** |  |  |
| **AT2G41820** |  |  |
| **AT2G42690** |  |  |
| **AT2G43800** |  | Pericycle |
| **AT2G46440** |  |  |
| **AT2G46450** |  |  |
| **AT2G46630** |  |  |
| **AT2G46690** |  | Pericycle |
| **AT2G47860** |  |  |
| **AT2G48080** |  | Early Differentiation Zone |
| **AT3G01670** |  |  |
| **AT3G01690** |  | Meristematic Zone |
| **AT3G01730** |  | Early Differentiation Zone |
| **AT3G02240** |  |  |
| **AT3G03190** |  |  |
| **AT3G03200** |  |  |
| **AT3G03450** | ***RGL2*** |  |
| **AT3G03550** |  |  |
| **AT3G04110** |  |  |
| **AT3G04630** | ***WDL1*** | Pericycle |
| **AT3G07410** |  | Meristematic Zone |
| **AT3G07420** |  |  |
| **AT3G07490** |  |  |
| **AT3G09070** |  |  |
| **AT3G10080** |  | Stele |
| **AT3G10120** |  |  |
| **AT3G11280** |  | Stele, Pericycle, Early Differentiation Zone |
| **AT3G12520** | ***SULTR4;2*** | Pericycle |
| **AT3G12700** |  | Early Differentiation Zone |
| **AT3G13380** | ***BRL3*** | QC/Initials, Pericycle |
| **AT3G13690** |  |  |
| **AT3G13730** | ***CYP90D1*** | Pericycle, Early Differentiation Zone |
| **AT3G13760** |  |  |
| **AT3G14060** |  |  |
| **AT3G14650** |  | Pericycle |
| **AT3G14660** |  | Pericycle, Early Differentiation Zone |
| **AT3G14690** |  |  |
| **AT3G14780** |  |  |
| **AT3G16920** |  | Early Differentiation Zone |
| **AT3G17380** |  |  |
| **AT3G17600** | ***IAA31*** |  |
| **AT3G18080** |  |  |
| **AT3G18260** |  |  |
| **AT3G18280** |  | Stele, Pericycle, Early Differentiation Zone |
| **AT3G19370** |  | Pericycle, Early Differentiation Zone |
| **AT3G21750** |  | QC/Initials |
| **AT3G24810** | ***KRP5*** |  |
| **AT3G25790** |  |  |
| **AT3G26210** |  |  |
| **AT3G26290** |  |  |
| **AT3G26830** | ***PAD3/CYP71B15*** | Pericycle |
| **AT3G27170** |  | Early Differentiation Zone |
| **AT3G27650** |  |  |
| **AT3G28130** |  |  |
| **AT3G29810** |  | Meristematic Zone |
| **AT3G44990** |  | Pericycle |
| **AT3G45160** |  |  |
| **AT3G45210** |  | Early Differentiation Zone |
| **AT3G45410** |  |  |
| **AT3G46500** |  |  |
| **AT3G46810** |  |  |
| **AT3G47180** |  |  |
| **AT3G47510** |  | Pericycle, Early Differentiation Zone |
| **AT3G47620** |  | Stele, Pericycle |
| **AT3G48080** |  |  |
| **AT3G48920** |  | Early Differentiation Zone |
| **AT3G48970** |  | Stele, Pericycle |
| **AT3G50070** | ***CYCD3;3*** | Meristematic Zone |
| **AT3G51350** |  |  |
| **AT3G52460** |  | Early Differentiation Zone |
| **AT3G52840** |  |  |
| **AT3G52900** |  | Stele, Early Differentiation Zone |
| **AT3G53200** |  |  |
| **AT3G54040** |  | Early Differentiation Zone |
| **AT3G55040** |  |  |
| **AT3G57140** |  |  |
| **AT3G57380** |  |  |
| **AT3G57780** |  |  |
| **AT3G58350** |  |  |
| **AT3G59680** |  | Stele |
| **AT3G60490** |  |  |
| **AT3G61220** |  | Pericycle |
| **AT3G61380** |  | Early Differentiation Zone |
| **AT3G63110** | ***AtIPT3*** |  |
| **AT4G00040** |  |  |
| **AT4G00680** |  | Early Differentiation Zone |
| **AT4G01110** |  |  |
| **AT4G02270** |  | Early Differentiation Zone |
| **AT4G03330** |  | Early Differentiation Zone |
| **AT4G08470** |  |  |
| **AT4G09020** |  | Meristematic Zone |
| **AT4G09100** |  | Early Differentiation Zone |
| **AT4G11230** |  |  |
| **AT4G11530** |  |  |
| **AT4G12270** |  |  |
| **AT4G12440** |  | Early Differentiation Zone |
| **AT4G12470** |  |  |
| **AT4G12490** |  |  |
| **AT4G12500** |  |  |
| **AT4G14060** |  |  |
| **AT4G14465** |  | Stele, Pericycle |
| **AT4G14630** |  |  |
| **AT4G14890** |  |  |
| **AT4G15530** |  | Meristematic Zone |
| **AT4G16260** |  |  |
| **AT4G16350** |  |  |
| **AT4G16920** |  |  |
| **AT4G16950** |  |  |
| **AT4G17550** |  | Pericycle |
| **AT4G18610** |  | Pericycle, Early Differentiation Zone |
| **AT4G18630** |  |  |
| **AT4G18640** |  | Early Differentiation Zone |
| **AT4G19460** |  | Meristematic Zone |
| **AT4G20270** |  | Stele, Pericycle, Early Differentiation Zone |
| **AT4G22080** |  | Early Differentiation Zone |
| **AT4G22270** |  |  |
| **AT4G25090** |  | Early Differentiation Zone |
| **AT4G25110** |  |  |
| **AT4G25790** |  | Early Differentiation Zone |
| **AT4G26260** |  |  |
| **AT4G27290** |  |  |
| **AT4G28720** |  |  |
| **AT4G30140** |  |  |
| **AT4G30320** |  | Early Differentiation Zone |
| **AT4G30560** |  | Early Differentiation Zone |
| **AT4G30640** |  |  |
| **AT4G31500** | ***SUR2*** | QC/Initials, Pericycle |
| **AT4G31730** | ***GDU1*** | Pericycle |
| **AT4G32860** |  |  |
| **AT4G33960** |  |  |
| **AT4G34580** |  | Early Differentiation Zone |
| **AT4G34950** |  |  |
| **AT4G34980** |  |  |
| **AT4G35970** |  |  |
| **AT4G36220** | ***FAH1/CYP84A1*** |  |
| **AT4G37295** |  | Pericycle |
| **AT4G37445** |  |  |
| **AT4G37550** |  | QC/Initials, Meristematic Zone |
| **AT4G37750** | ***ANT*** | Pericycle |
| **AT4G37890** |  |  |
| **AT4G37930** | ***SHM1*** | Pericycle, Early Differentiation Zone |
| **AT4G37970** |  | Pericycle |
| **AT4G38950** |  | Early Differentiation Zone |
| **AT4G38970** |  |  |
| **AT4G39190** |  | Pericycle |
| **AT4G39950** | ***CYP79B2*** | QC/Initials |
| **AT5G01780** |  |  |
| **AT5G02440** |  | Stele |
| **AT5G03050** |  |  |
| **AT5G03640** |  | Early Differentiation Zone |
| **AT5G03960** |  | Stele |
| **AT5G04310** |  |  |
| **AT5G04960** |  | Early Differentiation Zone |
| **AT5G05340** |  |  |
| **AT5G06330** |  |  |
| **AT5G06570** |  |  |
| **AT5G06800** |  |  |
| **AT5G07040** |  | Meristematic Zone |
| **AT5G07450** | ***CycP4;3*** | Early Differentiation Zone |
| **AT5G09460** |  |  |
| **AT5G10180** | ***Sultr2;1*** | Pericycle |
| **AT5G11930** |  |  |
| **AT5G14510** |  | Early Differentiation Zone |
| **AT5G14650** |  |  |
| **AT5G14760** |  |  |
| **AT5G17980** |  |  |
| **AT5G19800** |  | Early Differentiation Zone |
| **AT5G19970** |  |  |
| **AT5G20540** | ***BRXL4*** | Pericycle, Early Differentiation Zone |
| **AT5G20880** |  |  |
| **AT5G22875** |  |  |
| **AT5G23010** | ***MAM1*** |  |
| **AT5G23020** | ***MAM-L*** | Pericycle |
| **AT5G24010** |  | Pericycle, Early Differentiation Zone |
| **AT5G24313** |  | Early Differentiation Zone |
| **AT5G24780** |  |  |
| **AT5G25810** | ***TNY*** | Pericycle, Early Differentiation Zone |
| **AT5G25820** |  | Early Differentiation Zone |
| **AT5G25900** | ***GA3/CYP701A3*** | QC/Initials, Meristematic Zone |
| **AT5G33290** |  |  |
| **AT5G36270** |  | Early Differentiation Zone |
| **AT5G37990** |  |  |
| **AT5G38930** |  |  |
| **AT5G39190** |  |  |
| **AT5G40010** |  |  |
| **AT5G40860** |  | Early Differentiation Zone |
| **AT5G40910** |  |  |
| **AT5G41410** | ***BEL1*** |  |
| **AT5G42840** |  |  |
| **AT5G43150** |  | QC/Initials, Pericycle |
| **AT5G43370** |  | Early Differentiation Zone |
| **AT5G43590** |  |  |
| **AT5G44750** |  |  |
| **AT5G45380** | ***AtDUR3*** |  |
| **AT5G48290** |  | Early Differentiation Zone |
| **AT5G48500** |  |  |
| **AT5G49760** |  | Early Differentiation Zone |
| **AT5G49770** |  |  |
| **AT5G49870** |  |  |
| **AT5G51010** |  | Pericycle |
| **AT5G52390** |  |  |
| **AT5G53880** |  |  |
| **AT5G54040** |  | Early Differentiation Zone |
| **AT5G54790** |  |  |
| **AT5G55560** |  |  |
| **AT5G57130** |  | Stele |
| **AT5G57770** |  | Stele |
| **AT5G57780** |  |  |
| **AT5G58330** |  |  |
| **AT5G59530** |  |  |
| **AT5G59700** |  | Pericycle |
| **AT5G60200** |  | Stele, Pericycle |
| **AT5G60490** |  | Stele, Early Differentiation Zone |
| **AT5G61350** |  | Early Differentiation Zone |
| **AT5G61480** |  | Pericycle, Early Differentiation Zone |
| **AT5G61640** |  |  |
| **AT5G61650** | ***CYCP4;2*** | Early Differentiation Zone |
| **AT5G62710** |  |  |
| **AT5G65160** |  | Early Differentiation Zone |
| **AT5G65240** |  | Endodermis, Stele, Pericycle |
| **AT5G67400** |  | Early Differentiation Zone |
| **AT5G67430** |  |  |

The enrichment in the different tissues and longitudinal zones was calculated using the digital *in situ* data from (Birnbaum et al., 2003), (Nawy et al., 2005) and this work.

**References**

Birnbaum, K., Shasha, D. E., Wang, J. Y., Jung, J. W., Lambert, G. M., Galbraith, D. W., and Benfey, P. N. (2003). A gene expression map of the Arabidopsis root. Science *302*, 1956-1960.

Nawy, T., Lee, J. Y., Colinas, J., Wang, J. Y., Thongrod, S. C., Malamy, J. E., Birnbaum, K., and Benfey, P. N. (2005). Transcriptional profile of the Arabidopsis root quiescent center. Plant Cell *17*, 1908-1925.
