# Supplementary material for: Whole-Genome Analysis of the SHORT-ROOT Developmental Pathway in Arabidopsis
Source: PLoS Biol. 2006 May 2;4(5):e143. doi: 10.1371/journal.pbio.0040143 (PMC1450008; doi:10.1371/journal.pbio.0040143)
Supplement: Supplementary file 4 [file pbio.0040143.st002.doc]

**Table S2. Gene ontology analysis of SHR indirect** targets

| **GO main category** | **GO term** | **Number of genes annotated to the term** | **FDR**  **(False Positives)** | **Genes annotated to the term** |
| --- | --- | --- | --- | --- |
| **Indirect targets activated by SHR** | | |  |  |
| BP | [alcohol metabolism](http://godatabase.org/cgi-bin/go.cgi?query=GO:0006066) | 7 | 2% (0.06) | AT1G76550, AT5G40610, AT5G04120, AT1G17160, AT1G34430, AT2G29390, AT4G16155, |
| BP | [carboxylic acid metabolism](http://godatabase.org/cgi-bin/go.cgi?query=GO:0019752) | 10 | 1.6% (0.08) | AT3G04520, AT1G17160, AT1G24360, AT1G74960, AT3G15690, AT4G36250, AT5G08415, AT5G01320, AT5G47050, AT5G57350 |
|  |  |  |  |  |
| MF | [phosphoric monoester hydrolase activity](http://godatabase.org/cgi-bin/go.cgi?query=GO:0016791) | 4 | 3% (0.06) | AT1G17550, AT4G31750, AT5G59480, AT5G02230 |
| MF | [fatty-acid synthase activity](http://godatabase.org/cgi-bin/go.cgi?query=GO:0004312) | 2 | 2.67% (0.08) | AT1G24360, AT1G74960 |
|  |  |  |  |  |
| **Indirect targets repressed by SHR** | | |  |  |
| BP | [protein amino acid phosphorylation](http://godatabase.org/cgi-bin/go.cgi?query=GO:0006468) | 40 | 0% (0) | AT1G05700, AT1G11330, AT1G20650, AT1G21590, AT1G33260, AT1G49160, AT1G51170, AT1G51830, AT1G51860, AT1G51940, AT1G52310, AT1G61860, AT1G64630, AT1G67520, AT1G70460, AT1G76040, AT1G77280, AT1G79250, AT2G23030, AT2G25090, AT2G39360, AT2G41820, AT3G13380, AT3G13690, AT3G45410, AT4G08470, AT4G11530, AT4G18640, AT4G20270, AT5G03640, AT5G24010, AT4G27290, AT5G49760, AT5G49770, AT5G55560, AT5G59700, AT5G61350, AT5G61480, AT5G62710, AT5G65240 |
| BP | [response to biotic stimulus](http://godatabase.org/cgi-bin/go.cgi?query=GO:0009607) | 20 | 0.75% (0.06) | AT1G05250, AT1G05760, AT1G30870, AT1G59124, AT1G64160, AT1G71695, AT1G76930, AT2G02100, AT2G02130, AT2G18140, AT3G16920, AT3G26830, AT3G52460, AT4G16920, AT4G35970, AT5G05340, AT5G24780, AT5G36270, AT5G40910, AT5G67400 |
| BP | [glucosinolate biosynthesis](http://godatabase.org/cgi-bin/go.cgi?query=GO:0019761) | 3 | 0.67% (0.08) | AT1G16410, AT4G31500, AT5G23010 |
| BP | [macromolecule metabolism](http://godatabase.org/cgi-bin/go.cgi?query=GO:0043170) | 97 | 0.77% (0.1) | AT1G51830, AT1G21590, AT1G33970, AT5G33290, AT5G57130, AT2G24280, AT1G03310, AT1G03310, AT1G05190, AT1G05700, AT1G11330, AT1G14070, AT1G14720, AT1G16410, AT1G19650, AT1G20650, AT1G21590, AT1G22500, AT1G23160, AT1G33260, AT1G33970, AT1G48930, AT1G49160, AT1G49230, AT1G51170, AT1G51830, AT1G51860, AT1G51940, AT1G52310, AT1G61860, AT1G64630, AT1G67520, AT1G70460, AT1G72220, AT1G73270, AT1G76040, AT1G77020, AT1G77280, AT1G79250, AT2G03720, AT2G23030, AT2G24280, AT2G25090, AT2G26820, AT2G28720, AT2G32430, AT2G33770, AT2G34790, AT2G36870, AT2G39360, AT2G41820, AT2G43800, AT3G03550, AT3G07410, AT3G07420, AT3G07490, AT3G12700, AT3G13380, AT3G13690, AT3G18080, AT3G45410, AT3G46810, AT3G47180, AT3G51350, AT3G52840, AT4G08470, AT4G09020, AT4G09100, AT4G11530, AT4G15530, AT4G16260, AT4G18640, AT4G20270, AT4G25110, AT4G27290, AT4G31500, AT4G37890, AT4G38970, AT5G03640, AT5G07040, AT5G14650, AT5G23010, AT5G24010, AT5G33290, AT5G40010, AT5G45380, AT5G49760, AT5G49770, AT5G55560, AT5G57130, AT5G58330, AT5G59700, AT5G61350, AT5G61480, AT5G61640, AT5G62710, AT5G65240 |
|  |  |  |  |  |
| MF | [kinase activity](http://godatabase.org/cgi-bin/go.cgi?query=GO:0016301) | 42 | 0% (0) | AT1G05700, AT1G11330, AT1G20650, AT1G21590, AT1G33260, AT1G49160, AT1G51170, AT1G51830, AT1G51860, AT1G51940, AT1G52310, AT1G61860, AT1G67520, AT1G70460, AT1G76040, AT1G77280, AT1G79250, AT2G23030, AT2G25090, AT2G39360, AT2G41820, AT3G13380, AT3G13690, AT3G45410, AT4G08470, AT4G11530, AT4G15530, AT4G16920, AT4G18640, AT4G20270, AT4G27290, AT5G03640, AT5G24010, AT5G40910, AT5G49760, AT5G49770, AT5G55560, AT5G59700, AT5G61350, AT5G61480, AT5G62710, AT5G65240 |
| MF | [alpha-amylase activity](http://godatabase.org/cgi-bin/go.cgi?query=GO:0004556) | 2 | 0.4% (0.02) | AT1G03310, AT4G09020 |
| MF | [protein serine/threonine kinase activity](http://godatabase.org/cgi-bin/go.cgi?query=GO:0004674) | 18 | 1% (0.06) | AT1G11330, AT1G20650, AT1G21590, AT1G61860, AT1G67520, AT1G70460, AT1G76040, AT1G77280, AT2G23030, AT2G25090, AT3G13380, AT4G08470, AT4G20270, AT4G27290, AT5G24010, AT5G49770, AT5G61480, AT5G62710 |
| MF | [peroxidase activity](http://godatabase.org/cgi-bin/go.cgi?query=GO:0004601) |  | 0.86% (0.06) | AT1G05250, AT1G30870, AT1G71695, AT2G18140, AT4G35970, AT5G05340, AT5G67400 |
| MF | [2-isopropylmalate synthase activity](http://godatabase.org/cgi-bin/go.cgi?query=GO:0003852) | 3 | 0.67% (0.08) | AT5G23010, AT5G23020 |
| MF | [transcription factor activity](http://godatabase.org/cgi-bin/go.cgi?query=GO:0003700) | 34 | 0.62% (0.08) | AT1G02220, AT1G04550, AT1G19850, AT1G30490, AT1G30650, AT1G52880, AT1G57560, AT1G64630, AT1G66350, AT1G68810, AT1G68880, AT1G75410, AT1G77920, AT1G79580, AT2G25000, AT2G28350, AT2G29660, AT2G31380, AT2G34710, AT3G03200, AT3G03450, AT3G11280, AT3G17600, AT3G25790, AT3G47620, AT3G48920, AT3G53200, AT3G60490, AT4G37750, AT5G06800, AT5G09460, AT5G25810, AT5G41410, AT5G60200 |
| MF | [monooxygenase activity](http://godatabase.org/cgi-bin/go.cgi?query=GO:0004497) | 12 | 0.57% (0.08) | AT1G12200, AT1G16410, AT3G13730, AT3G14650, AT3G14660, AT3G14690, AT3G26210, AT3G26290, AT3G26830, AT4G28720, AT4G31500, AT4G39950 |

BP: biological process; MF: molecular function; FDR: false discovery rate.
