# Supplementary material for: Whole-Genome Analysis of the SHORT-ROOT Developmental Pathway in Arabidopsis
Source: PLoS Biol. 2006 May 2;4(5):e143. doi: 10.1371/journal.pbio.0040143 (PMC1450008; doi:10.1371/journal.pbio.0040143)
Supplement: Supplementary file 5 [file pbio.0040143.st003.doc]

**Table S3. SHR indirect targets encoding transcription factors**

| **Locus name** | **Gene Name** | **TF Family^1^** | **Tissue/Zone in which the gene is enriched** | **Function (references)** |
| --- | --- | --- | --- | --- |
| **Activated genes** |  |  |  |  |
| AT1G43160 | *RAP2.6* | AP2/EREBP |  |  |
| AT5G42700 |  | B3 |  |  |
| AT3G26744 | *ICE1* | bHLH |  | Resistance to cold (Chinnusamy et al., 2003) |
| AT1G06070 |  | bZIP |  |  |
| AT5G15150 | *AtHB3/HAT7* | HB | End |  |
| AT5G46910 |  | Jumonji |  |  |
| AT5G57620 |  | MYB |  |  |
| AT4G37610 | *AtBT5* | TAZ |  |  |
| AT1G51540 |  | putative DNA-binding protein | EZ |  |
| AT3G11930 |  | putative DNA-binding protein | End, Per, Ste, EDZ |  |
|  |  |  |  |  |
| **Repressed genes** |  |  |  |  |
| AT3G60490 |  | AP2/EREBP |  |  |
| AT4G37750 | *ANT* | AP2/EREBP | Per | Control of cell proliferation (Krizek, 1999; Mizukami and Fischer, 2000) |
| AT5G25810 | *TNY* | AP2/EREBP | Per, EDZ | Cell expansion (Wilson et al., 1996) |
| AT2G28350 | *ARF10* | ARF |  | Auxin signaling (Liscum and Reed, 2002) |
| AT1G19850 | *MP/ARF5* | ARF | MZ | Auxin signaling (Hardtke and Berleth, 1998) |
| AT1G31320 |  | AS2 |  |  |
| AT3G27650 |  | AS2 |  |  |
| AT3G17600 | *IAA31* | Aux/IAA |  | Auxin signaling (Liscum and Reed, 2002) |
| AT1G04550 | *BDL/IAA12* | Aux/IAA | Per, Ste | Auxin signaling (Hamann et al., 2002) |
| AT1G68810 |  | bHLH | Ste |  |
| AT5G09460 |  | bHLH |  |  |
| AT1G77920 |  | bZIP | EDZ |  |
| AT1G68880 |  | bZIP | Per |  |
| AT5G60200 |  | C2C2(Zn) | Per, Ste |  |
| AT2G31380 |  | C2C2(Zn) |  |  |
| AT2G29660 |  | C2C2(Zn) | Per |  |
| AT1G22500 |  | C3H (Zn) | EDZ |  |
| AT1G49230 |  | C3H (Zn) |  |  |
| AT1G72220 |  | C3H (Zn) | EDZ |  |
| AT3G03550 |  | C3H (Zn) |  |  |
| AT3G47180 |  | C3H (Zn) |  |  |
| AT4G09100 |  | C3H (Zn) | EDZ |  |
| AT4G37890 |  | C3H (Zn) |  |  |
| AT5G07040 |  | C3H (Zn) | MZ |  |
| AT3G25790 |  | G2-like |  |  |
| AT5G06800 |  | G2-like |  |  |
| AT1G66350 | *RGL1* | GRAS | MZ | Gibberellin signaling (Wen and Chang, 2002) |
| AT3G03450 | *RGL2* | GRAS |  | Gibberellin signaling (Lee et al., 2002) |
| AT1G30490 | *PHV* | HB |  | Vasculature development/organ polarity (McConnell et al., 2001) |
| AT1G75410 | *BLH3* | HB | EDZ |  |
| AT2G34710 | *PHB* | HB | MZ | Vasculature development/organ polarity (McConnell et al., 2001) |
| AT5G41410 | *BEL1* | HB |  | Ovule development (Reiser et al., 1995) |
| AT1G52880 |  | NAC |  |  |
| AT1G02220 |  | NAC |  |  |
| AT1G79580 |  | NAC | MZ |  |
| AT3G03200 |  | NAC |  |  |
| AT1G57560 |  | MYB | EDZ |  |
| AT3G11280 |  | MYB | Per, Ste, EDZ |  |
| AT3G48920 |  | MYB | EDZ |  |
| AT3G53200 |  | MYB |  |  |
| AT3G47620 |  | TCP | Per, Ste |  |
| AT1G70460 |  | Trihelix | EDZ |  |
| AT2G25000 |  | WRKY | EDZ |  |
| AT1G30650 | *WRKY14* | WRKY |  |  |
| AT2G18350 |  | ZF-HB |  |  |
| AT4G14465 |  | putative DNA-binding protein | Per, Ste |  |

**^1^** Families are according to (Riechmann, 2002), DATF (datf.cbi.pku.edu.cn) and the Sheen lab website (genetics.mgh.harvard.edu/sheenweb); TF: transcription factor. End: endodermis; Per: pericycle; Ste: stele; QC: quiescent center; MZ: meristematic zone; EZ: elongation Zone; EDZ: early differentiation zone. The enrichment in the different tissues and longitudinal zones was calculated using the digital *in situ* data from (Birnbaum et al., 2003), (Nawy et al., 2005) and this work.

**References**

Birnbaum, K., Shasha, D. E., Wang, J. Y., Jung, J. W., Lambert, G. M., Galbraith, D. W., and Benfey, P. N. (2003). A gene expression map of the Arabidopsis root. Science *302*, 1956-1960.

Chinnusamy, V., Ohta, M., Kanrar, S., Lee, B. H., Hong, X., Agarwal, M., and Zhu, J. K. (2003). ICE1: a regulator of cold-induced transcriptome and freezing tolerance in Arabidopsis. Genes Dev *17*, 1043-1054.

Hamann, T., Benkova, E., Baurle, I., Kientz, M., and Jurgens, G. (2002). The Arabidopsis BODENLOS gene encodes an auxin response protein inhibiting MONOPTEROS-mediated embryo patterning. Genes Dev *16*, 1610-1615.

Hardtke, C. S., and Berleth, T. (1998). The Arabidopsis gene MONOPTEROS encodes a transcription factor mediating embryo axis formation and vascular development. Embo J *17*, 1405-1411.

Krizek, B. A. (1999). Ectopic expression of AINTEGUMENTA in Arabidopsis plants results in increased growth of floral organs. Dev Genet *25*, 224-236.

Lee, S., Cheng, H., King, K. E., Wang, W., He, Y., Hussain, A., Lo, J., Harberd, N. P., and Peng, J. (2002). Gibberellin regulates Arabidopsis seed germination via RGL2, a GAI/RGA-like gene whose expression is up-regulated following imbibition. Genes Dev *16*, 646-658.

Liscum, E., and Reed, J. W. (2002). Genetics of Aux/IAA and ARF action in plant growth and development. Plant Mol Biol *49*, 387-400.

McConnell, J. R., Emery, J., Eshed, Y., Bao, N., Bowman, J., and Barton, M. K. (2001). Role of PHABULOSA and PHAVOLUTA in determining radial patterning in shoots. Nature *411*, 709-713.

Mizukami, Y., and Fischer, R. L. (2000). Plant organ size control: AINTEGUMENTA regulates growth and cell numbers during organogenesis. Proc Natl Acad Sci U S A *97*, 942-947.

Nawy, T., Lee, J. Y., Colinas, J., Wang, J. Y., Thongrod, S. C., Malamy, J. E., Birnbaum, K., and Benfey, P. N. (2005). Transcriptional profile of the Arabidopsis root quiescent center. Plant Cell *17*, 1908-1925.

Reiser, L., Modrusan, Z., Margossian, L., Samach, A., Ohad, N., Haughn, G. W., and Fischer, R. L. (1995). The BELL1 gene encodes a homeodomain protein involved in pattern formation in the Arabidopsis ovule primordium. Cell *83*, 735-742.

Riechmann, J. L. (2002). Transcriptional Regulation: a Genomic Overview. The Arabidopsis Book, 1-46.

Wen, C. K., and Chang, C. (2002). Arabidopsis RGL1 encodes a negative regulator of gibberellin responses. Plant Cell *14*, 87-100.

Wilson, K., Long, D., Swinburne, J., and Coupland, G. (1996). A Dissociation insertion causes a semidominant mutation that increases expression of TINY, an Arabidopsis gene related to APETALA2. Plant Cell *8*, 659-671.
