# Supplementary material for: Whole-Genome Analysis of the SHORT-ROOT Developmental Pathway in Arabidopsis
Source: PLoS Biol. 2006 May 2;4(5):e143. doi: 10.1371/journal.pbio.0040143 (PMC1450008; doi:10.1371/journal.pbio.0040143)
Supplement: Supplementary file 6 [file pbio.0040143.st004.doc]

**Table S4. SHR indirect** targets with kinase activity

| **Locus name** | **Family (subfamily^1^)** | **Gene name** | **Tissue/Zone in which the gene is enriched** | **References** |
| --- | --- | --- | --- | --- |
| **Activated by SHR** | |  |  |  |
| AT3G45430 | RLK (L-Lectin) |  |  |  |
| AT5G58300 | RLK (LRR-III) |  |  |  |
| AT5G63410 | RLK (LRR-VI) |  | End |  |
| AT3G14720 | MAPK | *AtMPK19* |  |  |
| AT1G17160 | pfkB-type carbohydrate kinase |  | EZ |  |
| AT1G76550 | 6-phosphofructose-1-kinase |  |  |  |
|  |  |  |  |  |
| **Repressed by SHR** | |  |  |  |
| AT1G52310 | RLK (C-Lectin) |  |  |  |
| AT2G39360 | RLK (CRPK1L-1) |  | EDZ |  |
| AT5G24010 | RLK (CRPK1L-1) |  | Per, EDZ |  |
| AT5G59700 | RLK (CRPK1L-1) |  | Per |  |
| AT5G61350 | RLK (CRPK1L-1) |  | EDZ |  |
| AT4G11530 | RLK (DUF26) |  |  |  |
| AT3G45410 | RLK (L-Lectin) |  |  |  |
| AT1G05700 | RLK (LRR-I) |  |  |  |
| AT1G51830 | RLK (LRR-I) |  |  |  |
| AT1G51860 | RLK (LRR-I) |  | EDZ |  |
| AT5G65240 | RLK (LRR-II) |  | End, Per, Ste |  |
| AT4G18640 | RLK (LRR-VI) |  | EDZ |  |
| AT5G49760 | RLK (LRR-VIII-1) |  | EDZ |  |
| AT5G49770 | RLK (LRR-VIII-1) |  |  |  |
| AT2G41820 | RLK (LRR-X) |  |  |  |
| AT3G13380 | RLK (LRR-X) | *BRL3* | Per, QC | (Cano-Delgado et al., 2004) |
| AT4G20270 | RLK (LRR-XI) |  | Per, Ste, EDZ |  |
| AT5G61480 | RLK (LRR-XI) |  | Per, EDZ |  |
| AT5G62710 | RLK (LRR-XIII) |  |  |  |
| AT1G51940 | RLK (LysM) |  |  |  |
| AT1G70460 | RLK (PERKL) |  | EDZ |  |
| AT3G13690 | RLK (PERKL) |  |  |  |
| AT1G21590 | RLK (RLCK-VI) |  | Per |  |
| AT1G77280 | RLK (RLCK-VI) |  |  |  |
| AT1G20650 | RLK (RLCKVII) |  | Per, EDZ |  |
| AT1G61860 | RLK (RLCKVII) |  | EDZ |  |
| AT1G11330 | RLK (SD-1) |  | Per |  |
| AT1G67520 | RLK (SD-1) |  |  |  |
| AT4G27290 | RLK (SD-1) |  |  |  |
| AT1G33260 | RLK |  |  |  |
| AT4G08470 | MAPKKK | *MAPKKK10* |  |  |
| AT1G76040 | CDPK | *CPK29* | End, Per |  |
| AT2G25090 | CIPK | *CIPK16* |  |  |
| AT1G49160 | WNK | *WNK7* |  |  |
| AT1G51170 | - |  | Per |  |
| AT1G64630 | - |  |  |  |
| AT1G79250 | - |  |  |  |
| AT2G23030 | - |  |  |  |

**^1^** RLK subfamilies are according to (Shiu and Bleecker, 2001; Shiu and Bleecker, 2003). End: endodermis; Per: pericycle; Ste: stele; QC: quiescent center; EZ: elongation Zone; EDZ: early differentiation zone. The enrichment in the different tissues and longitudinal zones was calculated using the digital *in situ* data from (Birnbaum et al., 2003), (Nawy et al., 2005) and this work.

**References**

Birnbaum, K., Shasha, D. E., Wang, J. Y., Jung, J. W., Lambert, G. M., Galbraith, D. W., and Benfey, P. N. (2003). A gene expression map of the Arabidopsis root. Science *302*, 1956-1960.

Cano-Delgado, A., Yin, Y. H., Yu, C., Vafeados, D., Mora-Garcia, S., Cheng, J. C., Nam, K. H., Li, J. M., and Chory, J. (2004). BRL1 and BRL3 are novel brassinosteroid receptors that function in vascular differentiation in Arabidopsis. Development *131*, 5341-5351.

Nawy, T., Lee, J. Y., Colinas, J., Wang, J. Y., Thongrod, S. C., Malamy, J. E., Birnbaum, K., and Benfey, P. N. (2005). Transcriptional profile of the Arabidopsis root quiescent center. Plant Cell *17*, 1908-1925.

Shiu, S. H., and Bleecker, A. B. (2001). Receptor-like kinases from Arabidopsis form a monophyletic gene family related to animal receptor kinases. Proc Natl Acad Sci U S A *98*, 10763-10768.

Shiu, S. H., and Bleecker, A. B. (2003). Expansion of the receptor-like kinase/Pelle gene family and receptor-like proteins in Arabidopsis. Plant Physiol *132*, 530-543.
