# Supplementary material for: Whole-Genome Analysis of the SHORT-ROOT Developmental Pathway in Arabidopsis
Source: PLoS Biol. 2006 May 2;4(5):e143. doi: 10.1371/journal.pbio.0040143 (PMC1450008; doi:10.1371/journal.pbio.0040143)
Supplement: Supplementary file 7 [file pbio.0040143.st005.doc]

**Table S5. SHR indirect targets involved in hormone-related processes**

| **Locus name** | **Gene name** | **Function** | **Tissue/Zone in which the gene is enriched** | **References** |
| --- | --- | --- | --- | --- |
| **Gibberellin** |  |  |  |  |
| AT1G66350 (R) | *RGL1* | Gibberellin signaling | MZ | (Wen and Chang, 2002) |
| AT3G03450 (R) | *RGL2* | Gibberellin signaling |  | (Lee et al., 2002) |
| AT5G25900 (R) | *GA3/CYP701A3* | Gibberellin biosynthesis | MZ | (Helliwell et al., 1998) |
|  |  |  |  |  |
| **Brassinosteroid** |  |  |  |  |
| AT3G20780 (A) | *TOP6B/BIN3/HYP6* | Brassinosteroid signaling |  | (Sugimoto-Shirasu et al., 2002; Yin et al., 2002) |
| AT3G13380 (R) | *BRL3* | Brassinosteroid perception | QC, Per | (Cano-Delgado et al., 2004) |
| AT3G13730 (R) | *Cyp90D1* | Brassinosteroid biosynthesis | Per, EDZ | (Kim et al., 2005) |
|  |  |  |  |  |
| **Auxin** |  |  |  |  |
| AT1G04550 (R) | *BDL/IAA12* | Auxin signaling | Per, Ste | (Hamann et al., 2002) |
| AT1G16410 (R) | *SPS/BUS/CYP79F1* | Auxin homeostasis modulator |  | (Reintanz et al., 2001; Tantikanjana et al., 2001) |
| AT1G19850 (R) | *MP/ARF5* | Auxin signaling | MZ | (Hardtke and Berleth, 1998) |
| AT1G23080 (R) | *PIN7* | Polar auxin transport |  | (Friml et al., 2003) |
| AT1G23160 (R) | *GH3-7* | Auxin signaling | MZ | (Hagen and Guilfoyle, 2002) |
| AT1G70940 (R) | *PIN3* | Polar auxin transport |  | (Friml et al., 2002) |
| AT2G28350 (R) | *ARF10* | Auxin signaling |  | (Liscum and Reed, 2002) |
| AT2G36210 (R) | *SAUR45* | Auxin signaling |  | (Hagen and Guilfoyle, 2002) |
| AT2G46690 (R) | *SAUR32* | Auxin signaling | Per | (Hagen and Guilfoyle, 2002) |
| AT3G17600 (R) | *IAA31* | Auxin signaling |  | (Liscum and Reed, 2002) |
| AT4G31500 (R) | *SUR2*/*CYP83B1* | Auxin homeostasis modulator | Per, QC | (Barlier et al., 2000) |
| AT4G39950 (R) | *CYP79B2* | Auxin homeostasis modulator | QC | (Ljung et al., 2005; Zhao et al., 2002) |

A: activated by SHR; R: repressed by SHR. Per: pericycle; Ste: stele; QC: quiescent center; MZ: meristematic zone; EDZ: early differentiation zone. The enrichment in the different tissues and longitudinal zones was calculated using the digital *in situ* data from (Birnbaum et al., 2003), (Nawy et al., 2005) and this work.

**References**

Barlier, I., Kowalczyk, M., Marchant, A., Ljung, K., Bhalerao, R., Bennett, M., Sandberg, G., and Bellini, C. (2000). The SUR2 gene of Arabidopsis thaliana encodes the cytochrome P450 CYP83B1, a modulator of auxin homeostasis. Proc Natl Acad Sci U S A *97*, 14819-14824.

Birnbaum, K., Shasha, D. E., Wang, J. Y., Jung, J. W., Lambert, G. M., Galbraith, D. W., and Benfey, P. N. (2003). A gene expression map of the Arabidopsis root. Science *302*, 1956-1960.

Cano-Delgado, A., Yin, Y. H., Yu, C., Vafeados, D., Mora-Garcia, S., Cheng, J. C., Nam, K. H., Li, J. M., and Chory, J. (2004). BRL1 and BRL3 are novel brassinosteroid receptors that function in vascular differentiation in Arabidopsis. Development *131*, 5341-5351.

Friml, J., Vieten, A., Sauer, M., Weijers, D., Schwarz, H., Hamann, T., Offringa, R., and Jurgens, G. (2003). Efflux-dependent auxin gradients establish the apical-basal axis of Arabidopsis. Nature *426*, 147-153.

Friml, J., Wisniewska, J., Benkova, E., Mendgen, K., and Palme, K. (2002). Lateral relocation of auxin efflux regulator PIN3 mediates tropism in Arabidopsis. Nature *415*, 806-809.

Hagen, G., and Guilfoyle, T. (2002). Auxin-responsive gene expression: genes, promoters and regulatory factors. Plant Mol Biol *49*, 373-385.

Hamann, T., Benkova, E., Baurle, I., Kientz, M., and Jurgens, G. (2002). The Arabidopsis BODENLOS gene encodes an auxin response protein inhibiting MONOPTEROS-mediated embryo patterning. Genes Dev *16*, 1610-1615.

Hardtke, C. S., and Berleth, T. (1998). The Arabidopsis gene MONOPTEROS encodes a transcription factor mediating embryo axis formation and vascular development. Embo J *17*, 1405-1411.

Helliwell, C. A., Sheldon, C. C., Olive, M. R., Walker, A. R., Zeevaart, J. A., Peacock, W. J., and Dennis, E. S. (1998). Cloning of the Arabidopsis ent-kaurene oxidase gene GA3. Proc Natl Acad Sci U S A *95*, 9019-9024.

Kim, G. T., Fujioka, S., Kozuka, T., Tax, F. E., Takatsuto, S., Yoshida, S., and Tsukaya, H. (2005). CYP90C1 and CYP90D1 are involved in different steps in the brassinosteroid biosynthesis pathway in Arabidopsis thaliana. Plant J *41*, 710-721.

Lee, S., Cheng, H., King, K. E., Wang, W., He, Y., Hussain, A., Lo, J., Harberd, N. P., and Peng, J. (2002). Gibberellin regulates Arabidopsis seed germination via RGL2, a GAI/RGA-like gene whose expression is up-regulated following imbibition. Genes Dev *16*, 646-658.

Liscum, E., and Reed, J. W. (2002). Genetics of Aux/IAA and ARF action in plant growth and development. Plant Mol Biol *49*, 387-400.

Ljung, K., Hull, A. K., Celenza, J., Yamada, M., Estelle, M., Normanly, J., and Sandberg, G. (2005). Sites and regulation of auxin biosynthesis in Arabidopsis roots. Plant Cell *17*, 1090-1104.

Nawy, T., Lee, J. Y., Colinas, J., Wang, J. Y., Thongrod, S. C., Malamy, J. E., Birnbaum, K., and Benfey, P. N. (2005). Transcriptional profile of the Arabidopsis root quiescent center. Plant Cell *17*, 1908-1925.

Reintanz, B., Lehnen, M., Reichelt, M., Gershenzon, J., Kowalczyk, M., Sandberg, G., Godde, M., Uhl, R., and Palme, K. (2001). Bus, a bushy Arabidopsis CYP79F1 knockout mutant with abolished synthesis of short-chain aliphatic glucosinolates. Plant Cell *13*, 351-367.

Sugimoto-Shirasu, K., Stacey, N. J., Corsar, J., Roberts, K., and McCann, M. C. (2002). DNA topoisomerase VI is essential for endoreduplication in Arabidopsis. Current Biology *12*, 1782-1786.

Tantikanjana, T., Yong, J. W., Letham, D. S., Griffith, M., Hussain, M., Ljung, K., Sandberg, G., and Sundaresan, V. (2001). Control of axillary bud initiation and shoot architecture in Arabidopsis through the SUPERSHOOT gene. Genes Dev *15*, 1577-1588.

Wen, C. K., and Chang, C. (2002). Arabidopsis RGL1 encodes a negative regulator of gibberellin responses. Plant Cell *14*, 87-100.

Yin, Y., Cheong, H., Friedrichsen, D., Zhao, Y., Hu, J., Mora-Garcia, S., and Chory, J. (2002). A crucial role for the putative Arabidopsis topoisomerase VI in plant growth and development. Proc Natl Acad Sci U S A *99*, 10191-10196.

Zhao, Y., Hull, A. K., Gupta, N. R., Goss, K. A., Alonso, J., Ecker, J. R., Normanly, J., Chory, J., and Celenza, J. L. (2002). Trp-dependent auxin biosynthesis in Arabidopsis: involvement of cytochrome P450s CYP79B2 and CYP79B3. Genes Dev *16*, 3100-3112.
