# Supplementary material for: Whole-Genome Analysis of the SHORT-ROOT Developmental Pathway in Arabidopsis
Source: PLoS Biol. 2006 May 2;4(5):e143. doi: 10.1371/journal.pbio.0040143 (PMC1450008; doi:10.1371/journal.pbio.0040143)
Supplement: Supplementary file 8 [file pbio.0040143.st006.doc]

**Table S6: Statistical Analysis of the Radial Zone Digital *in situ* Data.**

| SHR domain | | | | Non-SHR domain | | |  | | | |
| --- | --- | --- | --- | --- | --- | --- | --- | --- | --- | --- |
| Ste | Per | End | QC/I | Lrc | Col | Atr | Q < | Genes DE | Predicted False Positives | Genes enriched |
|  |  | X |  | X |  |  | 5.0 E-04 | 1609 | 0.80 | 875 |
|  |  | X |  |  | X |  | 2.5 E-04 | 4189 | 1.05 | 1591 |
|  |  | X |  |  |  | X | 5.0 E-04 | 1945 | 0.97 | 927 |
|  | X |  |  | X |  |  | 2.5 E-04 | 4354 | 1.09 | 2627 |
|  | X |  |  |  | X |  | 2.5 E-04 | 3491 | 0.87 | 1700 |
|  | X |  |  |  |  | X | 2.5 E-04 | 4859 | 1.21 | 2829 |
| X |  |  |  | X |  |  | 1.0 E-03 | 1011 | 1.01 | 564 |
| X |  |  |  |  | X |  | 2.5 E-04 | 6010 | 1.50 | 2430 |
| X |  |  |  |  |  | X | 1.0 E-03 | 1032 | 1.03 | 591 |
|  |  |  | X | X |  |  | 2.5 E-04 | 3067 | 0.77 | 2274 |
|  |  |  | X |  | X |  | 5.0 E-04 | 2361 | 1.48 | 1277 |
|  |  |  | X |  |  | X | 5.0 E-04 | 2963 | 1.48 | 2150 |

Radial tissue types were compared to each other to determine the number of genes with statistically significant differential expression (Genes DE) between layers in which SHR is present (SHR domain) and layers in which SHR is absent (Non-SHR domain). The q-value thresholds used are indicated (Q<) as well as the predicted number of false positives. In each line the tissues compared are indicated with crosses. The “Genes Enriched” column indicates the number of genes differentially expressed that are enriched in the layer from the SHR domain used in that given comparison. See Experimental Procedures for more details on the statistical analysis. Atr: atrichoblasts; Col: columella; End: endodermis; Lrc: lateral root cap; Per: pericycle; QC/I: QC and initials; Ste: stele.
